# Integrated multi-omics analysis reveals a glycolytic signature that predicts pan-cancer immune checkpoint inhibitor response and LDHA as a combinatorial target in fumarate hydratase-deficient renal cell carcinoma

**DOI:** 10.3389/fimmu.2025.1666121

**Published:** 2025-10-03

**Authors:** Songyang Liu, Yunlong Yuan, Junzhe He, Yihan Zhou, Yuan Wang, Xinqi Ye, Jianfeng Wang, Jin Zhang

**Affiliations:** ^1^ Department of Urology, Ren Ji Hospital, School of Medicine, Shanghai Jiao Tong University, Shanghai, China; ^2^ School of Medicine, Shanghai Jiao Tong University, Shanghai, China; ^3^ Department of Immunology, Medical College, Anhui University of Science and Technology, Huainan, Anhui, China; ^4^ State Key Laboratory of Natural Medicines, China Pharmaceutical University, Nanjing, Jiangsu, China; ^5^ Department of Urology, Renji Hospital, Shanghai Jiaotong University School of Medicine (Punan Hospital in Pudong New District, Shanghai), Shanghai, China

**Keywords:** FH-deficient RCC, immune checkpoint therapy, LdhA, glycolysis, single-cell sequencing, pan-cancer, large data analysis

## Abstract

**Introduction:**

Fumarate hydratase-deficient renal cell carcinoma (FH-deficient RCC) is a rare, aggressive malignancy with limited therapeutic options and poor prognosis. Despite immune checkpoint inhibitors (ICIs) showing efficacy in other cancers, responses in FH-deficient RCC remain suboptimal. Metabolic remodeling, particularly the Warburg effect-driven glycolysis, is implicated in immune evasion and tumor progression, highlighting the need for predictive biomarkers and combinatorial strategies.

**Methods:**

We integrated 41 single-cell RNA sequencing (scRNA-Seq) datasets (19 malignancies, 405 patients, 1,220,365 cells) to develop a glycolytic signature (Glyc.Sig). Validation included pan-cancer transcriptomic analysis (30 cancer types, n=10,154), CRISPR screening data (4 cancers), and clinical immunotherapy cohorts (5 cancers, n=921). LDHA was identified as a top-ranked immune-resistant candidate through CRISPR screening analysis, validated via immunoblotting and immunohistochemistry in Renji Hospital cohorts.

**Results:**

Glyc.Sig exhibited a robust inverse correlation between glycolytic activity and ICI efficacy across malignancies. It outperformed conventional biomarkers in predicting immunotherapy outcomes. CRISPR screening prioritized LDHA, a key glycolytic enzyme, as a target to enhance ICI response. Clinical validation confirmed elevated LDHA expression in FH-deficient RCC tumor tissues, which may correlate with immunosuppressive microenvironments and resistance to ICIs. Combinatorial LDHA inhibition and ICI treatment may demonstrate synergistic antitumor effects.

**Discussion:**

This study establishes Glyc.Sig as a dual diagnostic-predictive biomarker system, linking glycolytic reprogramming to immune evasion. Comparative validation revealed its enhanced predictive capacity for ICI responsiveness relative to existing molecular signatures. LDHA inhibition emerges as a promising strategy to overcome ICI resistance in FH-deficient RCC and other glycolytic tumors. These findings underscore the therapeutic potential of targeting cancer metabolism to optimize immunotherapy efficacy.

## Background

Fumarate hydratase-deficient renal cell carcinoma (FH-deficient RCC) represents a rare but clinically significant renal malignancy subtype, caused by functional inactivation of the fumarate hydratase (FH) gene (1). This aggressive tumor presents diagnostic complexities because of its non-specific histological features, requiring a comprehensive diagnostic approach. This includes immunohistochemical profiling that reveals the loss of FH protein alongside increased 2SC expression, complemented by molecular testing to confirm FH gene mutations (2). Despite its infrequent occurrence, FH-deficient RCC exhibits aggressive progression, often diagnosed at late stages (3–5). Those with advanced disease experience a poor prognosis, with a median survival of 18 to 24 months, highlighting the urgency for improved therapeutic strategies (4–6).

Immune checkpoint inhibitors (ICIs) have revolutionized oncology therapeutics, demonstrating unprecedented clinical efficacy across multiple malignancies (7). Emerging evidence has established that FH-deficient renal cell carcinoma features a highly immunogenic microenvironment. Clinical trials have shown that combining ICIs with tyrosine kinase inhibitors (TKIs) yields superior efficacy in patients with metastatic FH-deficient RCC compared to TKI monotherapy, with an objective response rate reaching 43.2% (8). However, a significant proportion of these cases still struggle to achieve sustained clinical remission. The persistent limitations of current therapeutic strategies, particularly the modest response rates observed in advanced disease stages, highlight the pressing need to develop robust predictive biomarkers. Such advancements would enable more precise patient selection and inform the design of optimized combination regimens targeting both immune evasion pathways and oncogenic signaling cascades.

Cancer cells undergo metabolic remodeling characterized by a predominant reliance on glycolysis to sustain survival and fulfill their biosynthetic/energetic demands (9). This adaptive strategy, known as the Warburg effect, not only confers proliferative advantages to malignant cells but also shapes an immunosuppressive tumor niche that facilitates cancer progression (10). Elevated glycolysis promotes lactate production, which acidifies the tumor microenvironment (TME), suppressing cytotoxic T-cell activity and fostering immunosuppressive cells like regulatory T cells (Tregs) and myeloid-derived suppressor cells (MDSCs) (11–13). Current prognostic models predominantly focus on single cancer types, while FH deficiency-induced metabolic dysregulation—the Warburg effect—is prevalent across multiple solid tumors. Thus, leveraging pan-cancer immunotherapy cohorts to construct cross-cancer glycolytic-related prognostic models may reveal universal biomarkers and provide an extrapolation validation basis for the rare FH-deficient RCC subtype.

**Figure 1:**
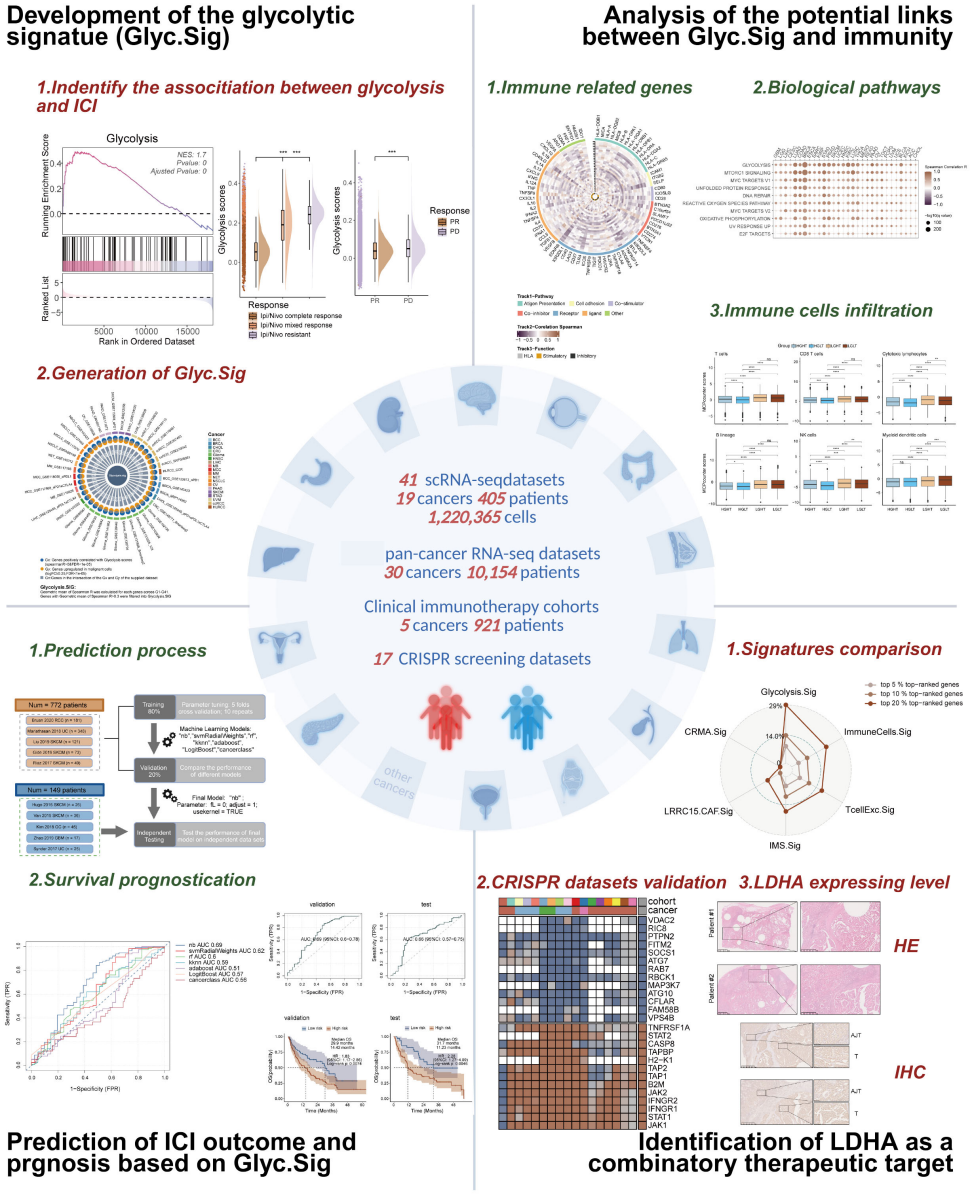
The graphical abstract of this study.

The primary objectives of this study are to investigate the role of tumor glycolytic activity in modulating the efficacy of ICIs and to develop a robust prognostic model based on glycolytic activity across various malignancies. We aim to identify a glycolytic signature (Glyc.Sig) that could serve as a universal biomarker for predicting ICIs’ efficacy in solid tumors, as well as uncover potential therapeutic targets to enhance ICIs’ responsiveness. By integrating scRNA-seq data from multiple cancer types, along with clinical immunotherapy outcomes, this study seeks to establish the Glyc.Sig as a predictive tool to guide therapeutic strategies. Additionally, functional CRISPR screening datasets will be explored to identify promising targets that can potentiate ICIs’ responsiveness across different malignancies. **Figure 1** presents the graphical abstract of this study.

## Materials and methods

### Bulk RNA analysis of FH-deficient RCC cohorts

Bulk RNA-seq data from a prior Renji Hospital study (14), including paired primary tumor and adjacent normal tissues from three FH-deficient RCC patients, were analyzed to assess differential gene expression patterns and their functional roles in glycolysis. Additionally, another FH-deficient RCC bulk RNA-seq cohort [GSE157256 dataset (15)] was adopted to investigate the different expression patterns across metastasis, primary tumors, and adjacent normal tissues.

### scRNA-seq ICI cohort analysis for glycolysis–immunotherapy links

To explore the associations between glycolysis and ICI response, four scRNA-seq datasets were evaluated: an FH-deficient RCC cohort [from a published article in the journal *Clin Cancer Res (* 16 *).*], a clear cell renal carcinoma (ccRCC) cohort [SRP308561 (17)], a skin cutaneous melanoma (SKCM) cohort [GSE115978 (18)], and a basal cell carcinoma (BCC) cohort (GSE123813 (19)). The obtained gene expression matrices were converted into Seurat objects, and all subsequent analyses were conducted using R software. Probable doublets were first removed using the DoubletFinder package. After integrating Seurat objects across all samples, strict quality control (QC) filters were applied to exclude cells meeting any of the following criteria: genes detected >7,500 or <200, total reads >75,000, or mitochondrial RNA content >20%. Qualifying cells underwent normalization and scaling via the LogNormalize method in Seurat, which concurrently identified highly variable genes. Principal component analysis (PCA) was performed on the highly variable gene matrix, with significant principal components (PCs) selected based on elbow plot inflection points and heatmap-driven evaluation of variance contributions. Using these PCs, a k-nearest neighbor graph was constructed (FindNeighbors), and graph-based clustering (FindClusters) was executed at a resolution parameter of 1. Cluster identities were visualized via UMAP projection, and cell annotations for all clusters were provided by the literature from the data sources.

Glycolysis-related genes from the Molecular Signatures Database (MSigDB) (https://www.gsea-msigdb.org/gsea/msigdb) were used to compute glycolysis pathway enrichment scores via gene set variation analysis (GSVA). Cohort details are summarized in **Supplementary Table S1**.

### Pan-cancer scRNA-seq datasets for glycolysis signature development

A pan-cancer glycolysis signature (Glyc.Sig) was developed using 41 scRNA-seq datasets (405 patients, 1,220,365 cells, 21 cancer types, **Supplementary Table S2**) encompassing malignant, stromal, and immune cells from the Gene Expression Omnibus (GEO, https://www.ncbi.nlm.nih.gov/geo/) and TISCH portal (http://tisch.comp-genomics.org/) (20), as well as directly from the published articles. These included breast cancer (BRCA), basal cell carcinoma (BCC), clear cell renal cell carcinoma (ccRCC), colorectal cancer (CRC), cholangiocarcinoma (CHOL), glioma, fumarate hydratase-deficient renal cell carcinoma (FH-deficient RCC), head and neck cancer (HNSC), liver hepatocellular carcinoma (LIHC), multiple myeloma (MM), Merkel cell carcinoma (MCC), medulloblastoma (MB), non-small cell lung cancer (NSCLC), neuroendocrine tumor (NET), ovarian serous cystadenocarcinoma (OV), pancreatic adenocarcinoma (PAAD), skin cutaneous melanoma (SKCM), stomach adenocarcinoma (STAD), and uveal melanoma (UVM) (16–19, 21–53).

### Pan-cancer transcriptomic analysis of the potential correlation between Glyc.Sig and immune suppression

TCGA pan-cancer transcriptomic data (10,154 patients; 30 cancer types) from the UCSC Xena data portal (https://xenabrowser.net) (54) were analyzed to evaluate the associations between Glyc.Sig and immune suppression. Acute myeloid leukemia (AML), diffuse large B-cell lymphoma (DLBC), and thymoma (THYM) were excluded due to immunocyte predominance (55). Tumor mutation burden (TMB) data from cBioPortal (https://www.cbioportal.org) (56, 57) and intratumor heterogeneity (ITH) data from a published article [Thorsson et al. (58)] were introduced to assess their correlations with Glyc.Sig scores calculated by GSVA.

### ICI RNA-seq cohorts for Glyc.Sig-based predictive modeling

Pretreatment transcriptomic data with clinical information from 10 ICI RNA-seq cohorts were used to develop and validate a Glyc.Sig-driven predictive model. These include five SKCM cohorts, provided by Van Allen in 2015 (59), Riaz in 2017 (60), Hugo in 2016 (61), Liu in 2019 (62), and Gide in 2019 (63); one renal cell carcinoma (RCC) cohort provided by Braun in 2020 (64); two urothelial carcinoma (UC) cohort from Mariathasan in 2018 (65) and Synder in 2017 (66); one glioblastoma multiforme (GBM) cohort from Zhao in 2019 (67); and one gastric cancer (GC) cohort from Kim in 2018 (68). Detailed cohort characteristics are demonstrated in **Supplementary Table S3**.

### CRISPR Screening for Immune Resistance Gene Identification

Seven CRISPR/Cas9 screening datasets derived from previous studies by Freeman (69), Kearney (70), Manguso (71), Pan (72), Patel (73), Vredevoogd (74), and Lawson (75) across multiple cancer types (BRCA, CRC, RCC, and SKCM) were analyzed to identify immune resistance-related genes. Following Fu et al. (76), who curated the first six datasets (except Lawson’s cohort), we divided the data from these 7 datasets into 17 distinct groups (**Supplementary Table S4**). The CRISPR screening methodological framework involved genome-wide CRISPR-Cas9 knockout in cancer cell lines subjected to cytotoxic lymphocyte (CTL) co-culture/not subjected to CTL (*in vitro*), or xenograft models in immune-deficient or immune-competent (*in vivo*) mice, followed by sgRNA abundance quantification through RNA sequencing (RNA-seq). Immunomodulating effects were quantified by calculating log-fold changes in sgRNA reads between experimental pairs (CTL-treated vs. untreated; immune-deficient vs. immune-competent mice) (75). Subsequent *Z*-score normalization was conducted for cross-dataset comparisons. Gene rankings were determined through an average *Z*-score across all datasets, and top-ranked genes with low *Z*-scores were regarded as potential mediators of immune resistance.

### Glycolysis scoring, pathway analysis, and immune profiling

GSVA (R package “GSVA”) was employed to calculate scores of HALLMARK pathways, Glyc.Sig, and glycolysis intensity (using glycolysis-related genes obtained from MsigDB). Pathway enrichment analysis was conducted using data from the Reactome Knowledgebase (https://reactome.org) and the Kyoto Encyclopedia of Genes and Genomes (KEGG) database (https://www.kegg.jp/) via the R package “clusterProfiler.” Immune infiltration was quantified using the R package MCP-counter v1.1.0.

### ICI response prediction model development

#### Cohort integration and preprocessing

Five RNA-seq cohorts, namely, Riaz 2017 SKCM (60), Mariathasan 2018 UC (65), Liu 2019 SKCM (62), Gide 2019 SKCM (63), and Braun 2020 RCC (64), which included 772 patients (181 RCC, 348 UC, and 243 SKCM patients), were merged. Batch effects were mitigated using the ComBat method (77). The cohort was split into training (80%, *n* = 618) and validation (20%, *n* = 154) sets, with an independent test set (*n* = 149) comprising five additional cohorts, including Van 2015 SKCM (59), Hugo 2016 SKCM (61), Snyder 2017 UC (66), Kim 2018 GC (68), and Zhao 2019 GBM (67).

#### Model optimization and validation

Seven machine learning algorithms, namely, AdaBoost Classification Trees (AdaBoost), boosted logistic regressions (LogitBoost), cancerclass, k-nearest neighbors (KNN), naive Bayes (NB), random forest (RF), and support vector machine (SVM) (78, 79), were trained with five-fold cross-validation (10 optimization iterations with different random seeds) (80). For cancerclass, the entire training set was used for model training, as cancerclass does not need parameters. Subsequently, we tested the performance of these models using the validation set. The top-performing model was selected for independent testing. Predicted risk stratification (“R” vs. “NR”) was evaluated by the final model for survival analysis.

#### Comparative signature analysis

Glyc.Sig was compared against six published ICI response signatures [INFG.Sig (81), T.cell.inflamed.Sig (81), PDL1.Sig (82), LRRC15.CAF.Sig (83), NLRP3.Sig (84), and Cytotoxic.Sig (85)] using individual AUC and average AUC values across 10 ICI cohorts. The algorithms and code for these six signatures were previously used in their original studies. Detailed information on these signatures is demonstrated in **Supplementary Table S5**.

### Immunohistochemistry

Immunohistochemical (IHC) staining was carried out on five tumor samples with adjacent normal tissues from FH-deficient RCC patients at Renji Hospital, with ethical committee approval. The experimental procedure included the following steps:

1. Paraffin sections underwent primary antibody incubation with LDHA (Proteintech 19987-1-AP, rabbit polyclonal Proteintech: Wuhan, China).
2. Subsequent application of peroxidase-conjugated goat anti-rabbit IgG secondary antibodies (Jackson ImmunoResearch: West Grove, Pennsylvania, USA 111-035-003).
3. Chromogenic detection using 3,3′-diaminobenzidine (DAB, Sigma-Aldrich: St. Louis, Missouri, USA D8001) coupled with hematoxylin counterstaining for nuclear visualization.

The final IHC score was determined by multiplying the scores for the percentage positivity of target protein-expressing cells.

### Immunoblotting analysis of paired tumor and adjacent tissues

FH-deficient RCC patients’ tumors and adjacent normal tissues were lysed in 2% SDS, followed by thermal denaturation at 99°C for 30 min. Proteins were separated by SDS-PAGE and then transferred onto nitrocellulose membranes. After blocking with 3% BSA in TBST for 1 h at room temperature, membranes were probed with primary antibodies through overnight incubation at 4°C. Following three washes with TBST, membranes were incubated with species-matched HRP-conjugated secondary antibodies (1:5,000 dilution) for 1 h at ambient temperature. Protein bands were visualized using chemiluminescence (Thermo Fisher Scientific). Paired samples from each patient were always run on the same gel to ensure comparability. Antibodies used in this study included anti‐LDHA (19987-1-AP, Proteintech) and anti‐β‐actin (66009-1-Ig, Proteintech).

### Statistical methods

Analyses were performed in R v4.3.1 (https://www.r-project.org). A two-sided Wilcoxon test was adopted to compare glycolysis scores between the ICI response and non-response groups. Spearman correlation analysis assessed associations between Glyc.Sig and other biological signatures, including scores of HALLMARK pathways, immune-related genes, ITH, TMB, and immune infiltration. FDR was adjusted via Benjamini–Hochberg. Model training, validation, and testing were conducted based on the R package cancerclass and caret. The predictive performance of the models was evaluated by ROC and AUC (86). Survival differences were analyzed using Cox regression analysis.

## Results

### Cancer glycolysis was linked to resistance to ICI

**Figure 2:**
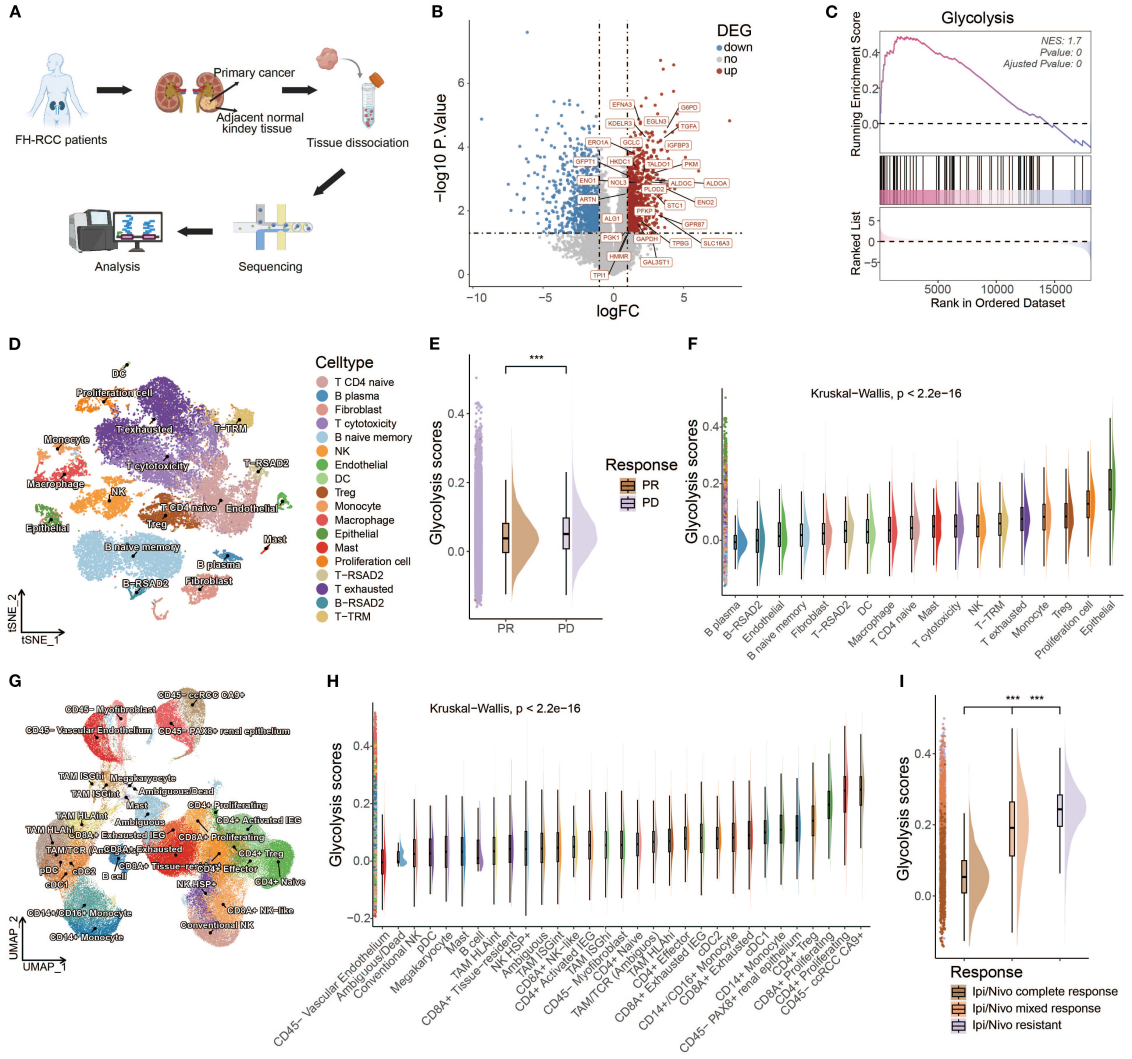
An inverse correlation was observed between glycolytic activity and response to ICIs in both FH-deficient RCC and ccRCC. **(A)** Schematic workflow of bulk RNA-seq analysis in FH-deficient RCC specimens. **(B)** Differential expression profile visualized through a volcano plot comparing FH-deficient RCC tumors with matched normal tissues. Significantly upregulated genes (red), downregulated genes (blue), and non-significant transcripts (gray) are demarcated. **(C)** Glycolytic pathway genes showed prominent enrichment through Gene Set Enrichment Analysis (GSEA) in FH-deficient RCC specimens. **(D)** Dimensionality reduction analysis using t-SNE visualization for FH-deficient RCC cellular populations. **(E)** Comparative distribution of glycolytic activity quantified through raincloud plots, stratified by treatment response categories (PR, partial response; PD, progressive disease) in FH-deficient RCC cases. **(F)** Cell type-specific glycolytic metabolic scores across FH-deficient RCC tumor microenvironments. **(G)** UMAP projection illustrating cellular heterogeneity in clear cell renal cell carcinoma (ccRCC) specimens. **(H)** Cellular compartment-based glycolysis quantification in ccRCC ecosystems. **(I)** Treatment response-associated glycolytic profiles (CR, complete response; MR, mixed response; R, resistance) depicted through raincloud plots for the ccRCC cohort. (*p < 0.05, **p < 0.01, ***p < 0.001).

FH-deficient RCC is an aggressive cancer syndrome driven by inactivation of the fumarate hydratase gene. To investigate the potential phenotype correlated with FH-deficient RCC, we utilized data from the Renji cohort in our prior investigation (14), which contains bulk RNA-seq data of primary cancer and adjacent normal tissues from three FH-deficient RCC patients (**Figure 2A**). After that, we compared the expression levels of different genes between cancer and adjacent normal tissues (**Figure 2B**), and some glycolysis-related genes, such as ENO1, PLOD2, and PKM, were upregulated. Considering loss-of-function mutations in FH trigger a metabolic crisis characterized by defective tricarboxylic acid (TCA) cycle flux, forcing cellular metabolic reprogramming toward aerobic glycolysis, we speculated that glycolysis might be more active in FH-deficient RCC cancer tissues. Therefore, we conducted the GSEA analysis of glycolysis pathways, and predictably, there was a significant positive enrichment of glycolysis pathways in cancer tissues of FH-deficient RCC (**Figure 2C**). Next, we investigated the relationship between glycolysis and ICI outcomes in FH-deficient RCC. **Figure 2D** showed the t-Distributed Stochastic Neighbor Embedding (t-SNE) visualization of the FH-deficient RCC from previously published datasets. Patients with progressive disease (PD) exhibited significantly higher levels of glycolysis compared to those with partial response (PR) (**Figure 2E**). In particular, glycolysis was more enriched in cancer cells (epithelial cells in **Figure 2F**) compared to other cell types. As glycolysis is a key metabolic pathway that is often upregulated in many cancers (87), we examined the findings above in another renal cancer type, clear cell renal cell carcinoma (ccRCC), and similar findings were observed, as shown in **Figures 2G–I**, further supporting the potential role of glycolysis in influencing the outcomes of ICI therapy.

**Figure 3:**
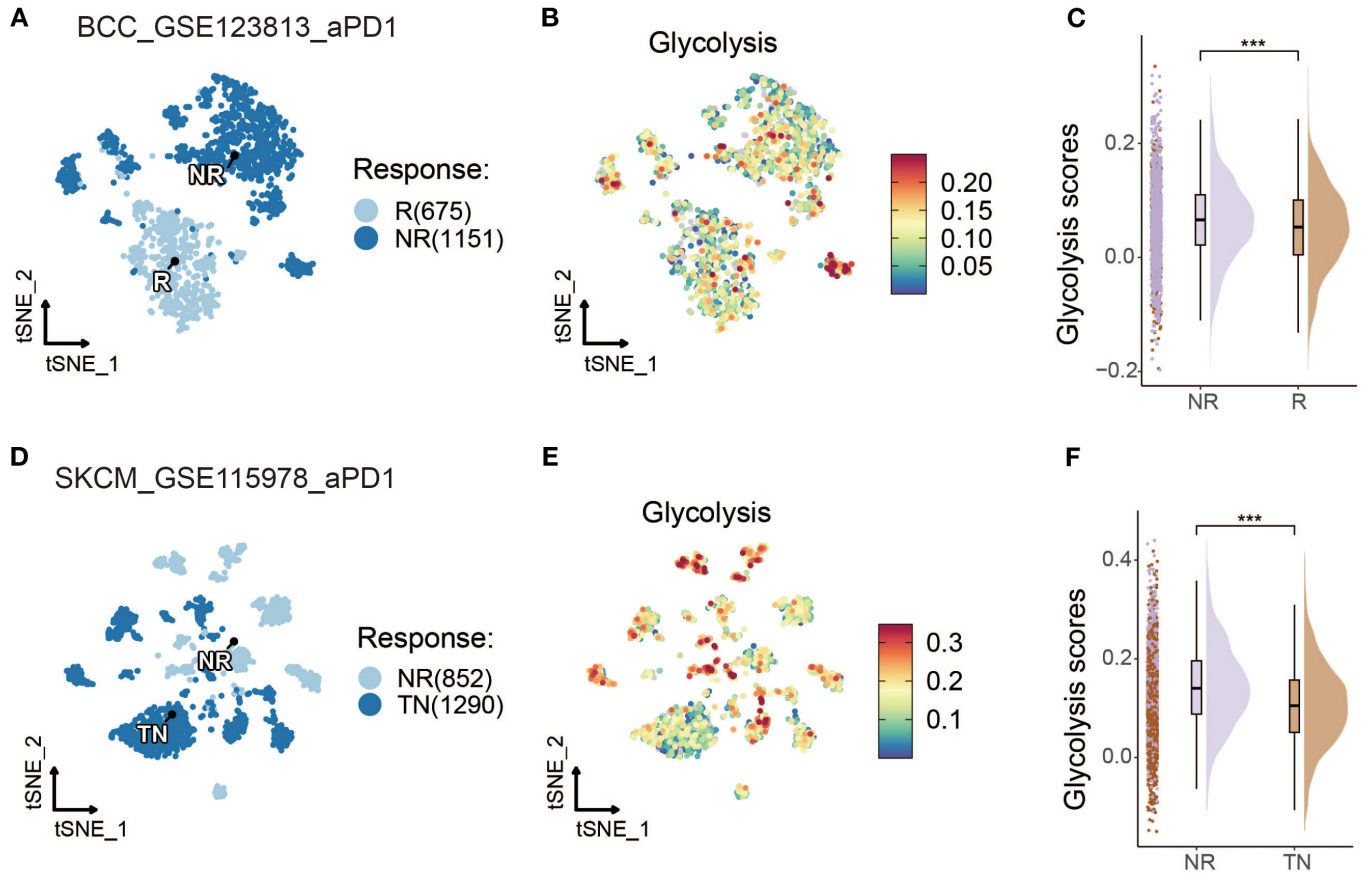
Glycolysis intensity was negatively correlated with ICI outcomes in other cancer types. **(A, D)** t-SNE map of BCC and SKCM malignant cells classified by treatment response status. **(B, E)** t-SNE visualization of BCC and SKCM malignant cells with dark-blue and dark-red indicating low and high glycolytic scores, respectively. **(C, F)** Comparative raincloud distribution analysis of glycolytic scores between non-responders (NR) and responders (R) or treatment-naive (TN) patients in BCC and SKCM specimens. (*p < 0.05, **p < 0.01, ***p < 0.001).

To further investigate the role of glycolysis in ICI responses, the relationships between ICI outcomes and glycolysis intensity were explored in BCC and SKCM cancer datasets. In BCC, **Figures 3A, B** demonstrate that non-responders (NR) exhibited a higher glycolysis intensity compared to responders (R). A quantitative analysis shown in **Figure 3C** further confirms this finding, revealing that the NR subgroup had significantly higher glycolysis intensity than the R subgroup. For SKCM, an additional dataset was used (18), which excluded samples lacking data on malignant cells, to validate the findings. However, due to missing data for some responders, the comparison was made between treatment-naive (TN) patients and NR. It was hypothesized that treatment-naive patients might consist of both potential responders and non-responders. The analysis also demonstrated a significantly higher glycolysis intensity in the NR subgroup than the TN subgroup in the SKCM cohort, as shown in **Figures 3D–F** (*p* < 0.001).

### Development of Glyc.Sig based on pan-cancer scRNA-seq datasets

**Figure 4:**
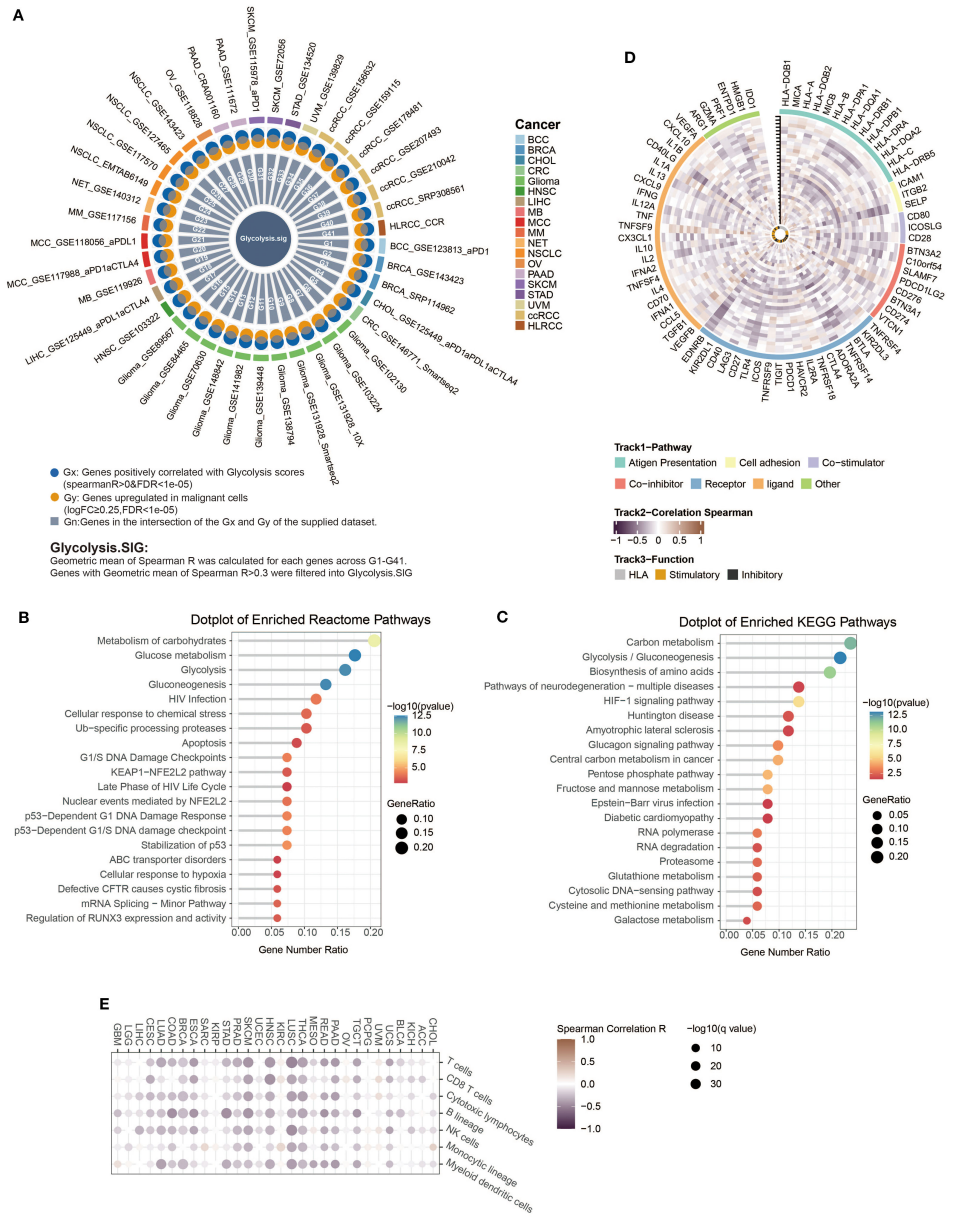
Construction and functional description of Glyc.Sig. **(A)** A circos diagram illustrated the developmental workflow of Glyc.Sig construction. **(B, C)** Functional pathway enrichment analysis was performed on genes in Glyc.Sig. **(B)** The top 20 enriched pathways based on the Reactome database; **(C)** the top 20 significantly enriched KEGG pathways. **(D)** Circos visualization revealing correlations between Glyc.Sig and immune-related gene expressions across various malignant tumors. The vertical tick-marked line denoted distinct cancer types, corresponding to the *x*-axis in **(E)**. **(E)** Heatmap visualization illustrating the relationships between Glyc.Sig and immune cell infiltration levels in multiple cancer types.

Since the intensity of glycolysis was significantly correlated with ICI resistance, we suggested that a gene set containing genes reflecting the level of glycolysis, named as glycolysis signature (Glyc.Sig), might help in predicting the ICI response. Therefore, we utilized 41 pan-cancer scRNA-seq datasets to develop the Glyc.Sig. Spearman correlation was initially adopted between enrichment scores of tumor glycolysis and gene expression levels. The Gx gene set contained genes with a positive correlation with scores of glycolysis intensity (*R* > 0 and FDR < 1e−05). The Gy gene set comprised significantly overexpressed genes in malignant cells. We intersected Gx and Gy to formulate the Gn gene set within each dataset (*n* = 1, 2,…, 41) (18), which contained upregulated genes of tumor specificity with a positive correlation with glycolysis intensity. For each gene in the G1–G41 gene sets, we calculated the geometric mean of the Spearman *R* value, and genes with a final *R* mean greater than 0.3 (88) were selected to form Glyc.Sig. The detailed gene list is demonstrated in **Supplementary Table S6**. The simplified process of the generation of Glyc.Sig could be intuitively observed in **Figure 4A**. Additionally, pathway analysis based on Reactome (**Figure 4B**) and KEGG (**Figure 4C**) was adopted to explore the biological functions of Glyc.Sig. The overrepresented pathways were primarily associated with glycolysis, hypoxia, the KEAP1-NFE2L2 pathway, G1/S DNA damage checkpoints, glutathione metabolism, and carbohydrate metabolism processes.

### Investigating potential correlations between Glyc.Sig and immunity

We first investigated the relations between Glyc.Sig and 75 immune-related genes (58), consisting of the HLA set, stimulatory set, and inhibitory set. A general negative correlation was found across almost all 75 genes in 30 different cancer types (**Figure 4D**). **Figure 4E** demonstrates the situation of immune cell infiltration. A higher level of glycolysis intensity was negatively correlated with various categories of immune cell infiltration, including cytotoxic cells (CD8 T cell, cytotoxic lymphocyte, NK cells), B lineage cells, and myeloid dendritic cells. The analysis above suggested a decreased antitumor immunity in tumors with a high intensity of glycolysis.

**Figure 5:**
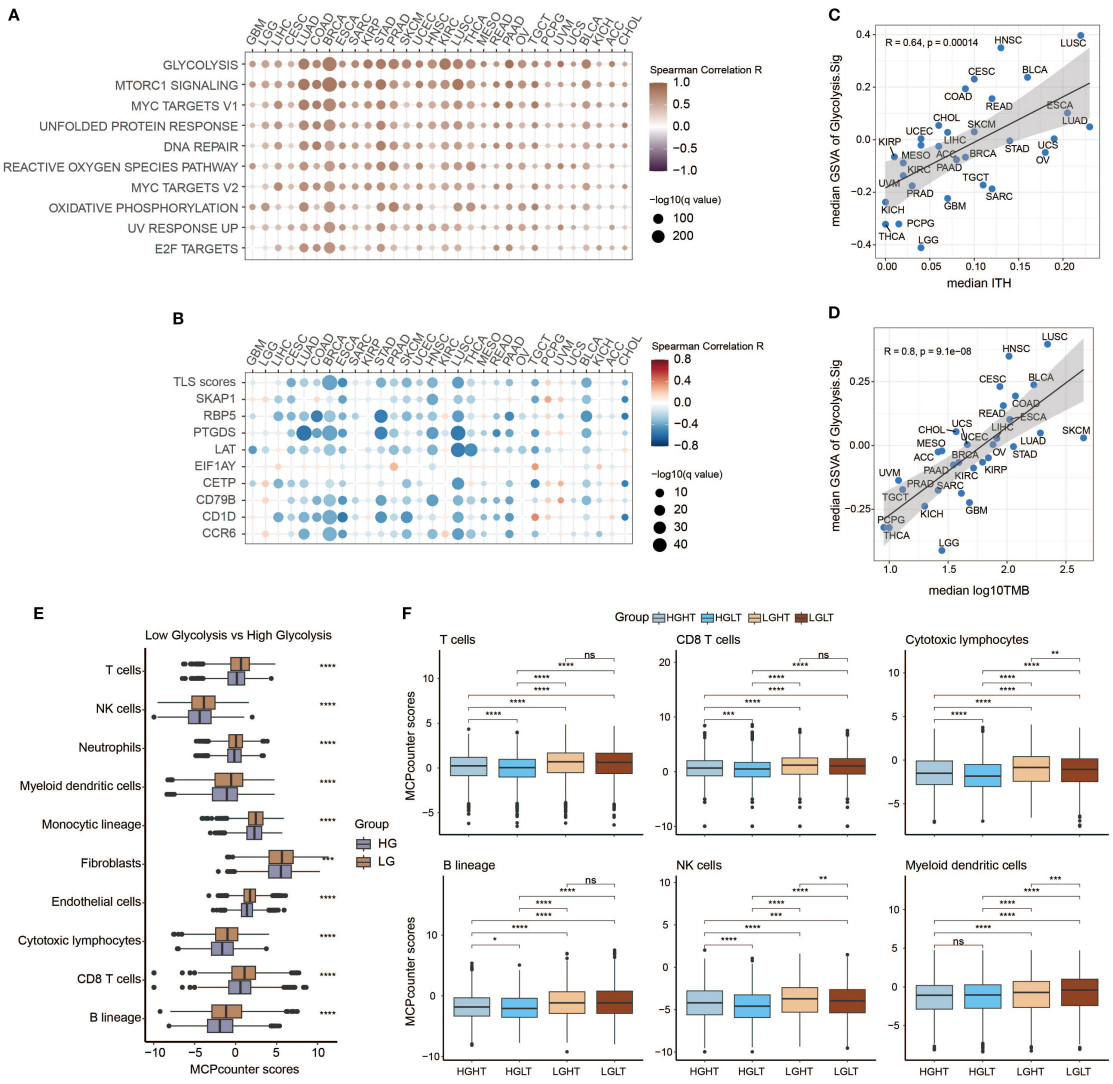
Investigation of potential relations between Glyc.Sig and immune resistance. **(A)** Heatmap illustrating the relationships between Glyc.Sig and the top 10 HALLMARK signaling pathways. **(B)** Heatmap depicting the relationships between Glyc.Sig and TLS-related genes. **(C)** Scatter plot showing the association between median GSVA scores of Glyc.Sig and median intratumor heterogeneity (ITH) across pan-cancer datasets. **(D)** Correlation analysis of median GSVA scores of Glyc.Sig with median log10 tumor mutational burden (TMB) levels in pan-cancer datasets. **(E)** Comparative box plots analyzing the relationship between MCP levels and Glyc.Sig activity. **(F)** Box plots evaluating specific immune cell infiltration patterns in groups with different Glyc.Sig status and TMB (Mann–Whitney *U* test; ns, not significant; *p < 0.05, **p < 0.01, ***p < 0.001, ****p < 0.0001.

Next, we explored the links between enrichment of HALLMARK pathways and expression intensity of Glyc.Sig. Both of them were calculated through GSVA. All of the top 10 HALLMARK pathways, including DNA repair, MYC signaling, and glycolysis, were positively correlated with the expression intensity of Glyc.Sig (**Figure 5A**). All of these pathways might be associated with a weak immune response based on previous studies (89–91). We also explored the relations between enrichment of tertiary lymphoid structure (TLS)-related genes and the expression intensity of Glyc.Sig. As shown in **Figure 5B**, TLS scores as well as most of the TLS-related genes were negatively related to the expression intensity of Glyc.Sig. Notably, high TLS scores and related genes usually indicated an abundant immune cell infiltration. Additionally, we investigated the relations between the median score of Glyc.Sig and the median ITH as well as the median TMB. ITH was a feature that facilitated immunosuppression (92). As anticipated, the expression intensity of Glyc.Sig was positively correlated with ITH (*R* = 0.64, *p* = 0.00014, **Figure 5C**). However, a positive link between TMB and Glyc.Sig was found (*R* = 0.8, *p* = 9.1e−8, **Figure 5D**), which seemed to go against our current understanding of TMB. Generally, higher TMB means better immune response because of abundant antigenicity, while a high level of Glyc.Sig does the opposite. Based on the median GSVA score of Glyc.Sig and the median TMB as grouping criteria, patients were divided into four distinct subgroups: high Glyc.Sig/high TMB (HGHT), high Glyc.Sig/low TMB (HGLT), low Glyc.Sig/high TMB (LGHT), and low Glyc.Sig/low TMB (LGLT). First, we clarified that both high Glyc.Sig and low TMB had decreased cytotoxic lymphocyte infiltration (**Figure 5E**, **Supplementary Figure S1A**), which was consistent with our current understanding. We further compared immune cell infiltration among the LGLT, LGHT, HGLT, and HGHT subgroups. LGHT was identified as the group with the highest infiltration of cytotoxic lymphocytes and NK cells, while HGLT was found to have the lowest infiltration of most immune cells, including cytotoxic lymphocytes, monocytic lineage, CD8 T cells, NK cells, T cells, and B lineage (**Figure 5F**, **Supplementary Figure S1B**). As for the LGLT and HGHT subgroups, the situation seemed to be unclear, possibly for the reason that these two subgroups had both immune-stimulating factors and inhibiting factors.

### Predicting immunotherapy response based on Glyc.Sig

**Figure 6:**
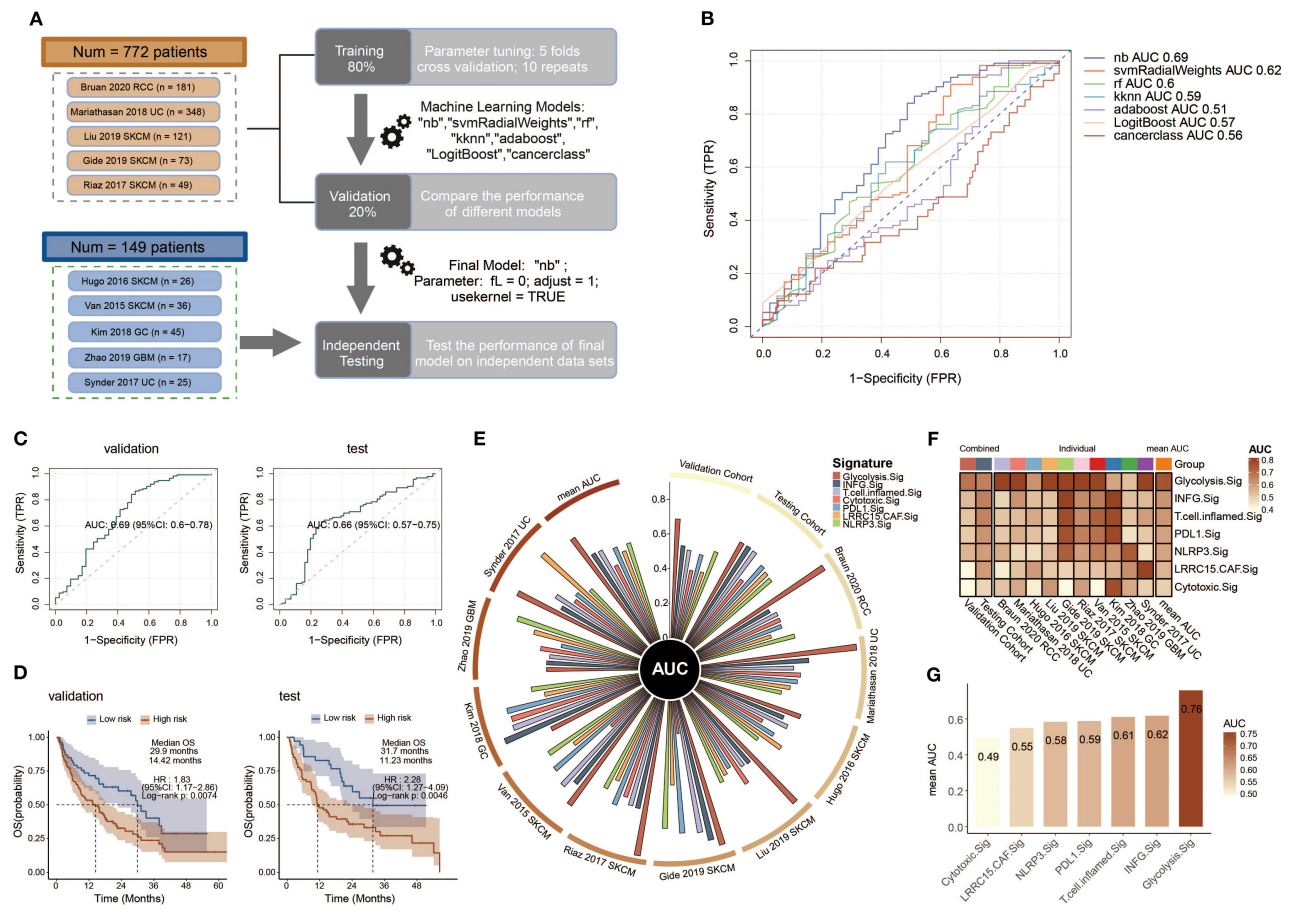
Construction and validation of a Glyc.Sig-based ICI outcome prediction model. **(A)** The workflow for developing the Glyc.Sig model encompassed training, validation, and testing phases, as demonstrated in the flowchart. The training stage incorporated five-fold cross-validation to optimize parameters among multiple machine learning approaches. During validation, the naive Bayes (nb) algorithm, demonstrating the best AUC performance, was chosen as the finalized Glyc.Sig model (parameters: fL = 0, adjust = 1, usekernel = TRUE). **(B)** Predictive accuracy assessment of diverse machine learning algorithms in the validation cohorts, quantified through AUC values. **(C)** Receiver operating characteristic (ROC) curves illustrating the classification performance of the final Glyc.Sig model (nb algorithm) across both validation and independent testing cohorts. **(D)** Comparative survival outcomes between model-predicted high-risk (non-responders) and low-risk (responders) subgroups, visualized through Kaplan–Meier survival curves in the validation and testing datasets. **(E)** Circos plot visualization of pan-cancer signature efficacy in different cohorts, with the vertical axis representing AUC measurements. **(F)** Comparative heatmap displaying predictive performance (AUC values) between Glyc.Sig and established pan-cancer signatures. **(G)** Bar chart visualization comparing AUC values of Glyc.Sig against other pan-cancer signatures. TPR, true positive rate; FPR, false positive rate; AUC, area under the curve; HR, hazard ratio; CI, confidence interval.

To explore the prognostic value of Glyc.Sig, bulk RNA-seq data, and the corresponding clinical data from 10 ICI cohorts were curated and analyzed to construct the models for ICI response prediction based on Glyc.Sig. To better utilize these data, we divided them into three subsets: the training set for model training and parameter tuning based on five-fold cross-validation repeated 10 times (except cancerclass); a validation set for model comparison and selection according to AUC; and the independent testing set for final model diagnosis. The flowchart reflected the whole process (**Figure 6A**). Seven different models were incorporated and trained at the beginning; naive Bayes was finally chosen as the Glyc.Sig model with the highest AUC of 0.69 (**Figure 6B**). Next, we utilized the independent testing set to make further evaluation of the Glyc.Sig model by predicting the ICI response. An AUC of 0.66 was finally observed (**Figure 6C**).

To evaluate the predictive value of Glyc.Sig on overall survival (OS), patients were divided into a high-risk group (predicted non-responders, NR) and a low-risk group (predicted responders, R) according to the prediction of ICI response given by the Glyc.Sig model. Kaplan–Meier survival analysis demonstrated that in both validation and testing cohorts, a high-risk group had a significantly shorter OS than the low-risk group (*p* < 0.01, **Figure 6D**). In the validation cohorts, the low-risk group exhibited a median OS of 29.9 months (HR = 1.83, 95% CI 1.17–2.86), nearly doubling the OS (14.42 months) observed in high-risk counterparts. In the testing cohort, low-risk patients had a 31.7-month median OS, while the OS in high-risk subjects was 11.23 months (HR = 2.28, 95% CI: 1.27–4.09). Subsequently, we examined the performance of the Glyc.Sig predictive model in each of the 10 ICI RNA-seq cohorts. The AUC across these 10 cohorts ranged from 0.53 to 0.91 (**Supplementary Figure S2**), with the AUC of the SKCM cohort in 2017 by Riaz reaching the highest (AUC = 0.91, 95% CI: 0.82–0.99), followed by a UC cohort in 2018 by Mariathasan (AUC = 0.90, 95% CI: 0.87–0.94). The GBM cohort in 2019 by Zhao exhibited the lowest predictive accuracy (AUC = 0.53, 95% CI: 0.24–0.82), which could be attributed to its restricted sample size. As for survival analysis, the GC cohort in 2018 by Kim was excluded due to the lack of OS data. For the remaining nine cohorts, high-risk patients predicted by the Glyc.Sig model showed significant survival benefits. Notable observations were made in the SKCM cohort from 2015 (Van Allen), the UC cohort from 2017 (Synder), the UC cohort from 2018 (Mariathasan), the SKCM cohort from 2019 (Liu), and the SKCM cohort from 2019 (Gide) (**Supplementary Figure S3**).

Comparison between Glyc.Sig and other well-constructed predictive gene signatures was made by calculating the AUC for each gene signature. In comparison with other gene signatures (81–85), Glyc.Sig performed best in the validation cohort (**Figure 6E**) with an AUC of 0.69. Additionally, Glyc.Sig manifested the best performance in most of the 10 individual ICI cohorts, while most of the other signatures performed ideally in only one or two cohorts (**Figure 6F**, **Supplementary Table S7**). We also compared the average AUC of these gene signatures over the 10 individual ICI cohorts. Notably, Glyc.Sig dominated the list with an average AUC of 0.76, surpassing the second signature (INFG.sig) with an AUC of 0.62 (**Figure 6G**).

### Investigation of potential therapeutic targets based on Glyc.Sig

**Figure 7:**
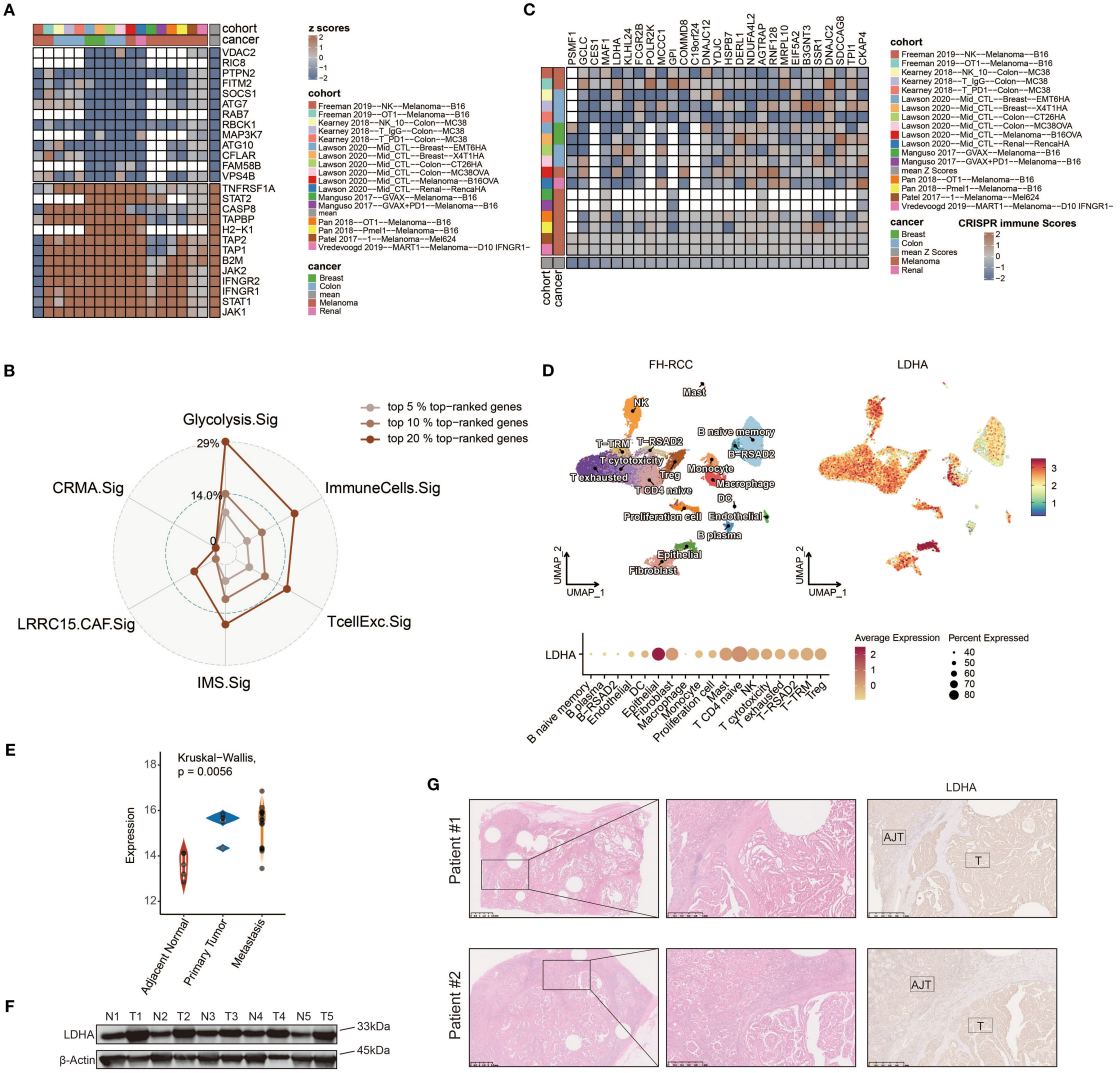
Identifying LDHA as a potential therapeutic target for FH-deficient RCC using CRISPR screening data. **(A)** Gene prioritization based on knockout effects on antitumor immune responses across the CRISPR subgroups. The classification was determined through aggregated *Z*-score computations, with elevated ranks signifying greater contributions to immune resistance. Vacancies in the heatmap reflect the absence of gene-level data in primary experimental cohorts. **(B)** Radar chart demonstrating the relative representation frequency of Glyc.Sig-associated genes within the 5%, 10%, and 20% top-ranked genes derived from the CRISPR datasets, compared with other predictive signatures. **(C)** *Z*-score visualization matrix depicting 27 Glyc.Sig genes ranked within the top 20% top-ranked genes derived from the CRISPR datasets. **(D)** Uniform manifold approximation and projection (UMAP) plot of FH-deficient RCC sample labeled by expression levels of LDHA. **(E)** Comparison among the expression levels of LDHA in metastasis lesion, primary lesion, and adjacent normal tissue in FH-deficient RCC. **(F)** Immunoblotting analysis of LDHA expression in FH-deficient RCC and adjacent normal tissues. **(G)** Representative IHC staining images of LDHA in the tumor and adjacent normal tissue areas.

Immune response data corresponding to gene knockouts were collected from seven CRISPR cohorts. These cohorts were further categorized into 17 datasets based on the model cells and treatments adopted. A total of 22,505 genes were documented across these datasets. By ranking the genes according to their average *Z*-scores, we identified the top-ranked genes as immune-resistant, suggesting that their knockout could enhance antitumor immunity. Conversely, the bottom-ranked genes were classified as immune-sensitive, indicating that their knockout might suppress antitumor immunity. The ranking process is depicted in **Figure 7A**. Out of the 22,505 genes, the numbers of genes in the top 5%, 10%, and 20% rankings were 1,125, 2,250, and 4,501, respectively. To further investigate the immunotherapeutic value of Glyc.Sig, we subsequently calculated and compared the proportion of the top 5%, 10%, and 20% ranked genes in Glyc.sig and other previously reported immune-resistant gene signatures, including TcellExc.Sig, ImmuneCells.Sig, IMS.Sig, LRRC15.CAF.Sig, and CRMA.Sig (18, 83, 93–95). The IPRES signature was excluded due to its unique composition of pathways rather than genes. As anticipated, Glyc.Sig had the highest proportion of overlapped top-ranked genes compared to other gene signatures (**Figure 7B**). The immune-resistant genes (those in the top 20%) were significantly enriched in Glyc.Sig (*p* = 0.02, Fisher’s exact test). Twenty-seven genes in Glyc.Sig, including PSMF1, GCLC, CES1, MAF1, LDHA, KLHL24, FCGR2B, POLR2K, MCCC1, GPI, COMMD8, C19orf24, DNAJC12, YDJC, HSPB7, DERL1, NDUFA4L2, AGTRAP, RNF128, MRPL10, EIF5A2, B3GNT3, SSR1, DNAJC2, SDCCAG8, TPI1, and CKAP4, ranked in the top 20%. The relations between tumor immune regulation and these glycolysis-related genes were verified using a series of independent CRISPR datasets (**Figure 7C**, **Supplementary Figure S4**), indicating their potential as therapeutic targets for combination with immune checkpoint blockade (ICB).

Given the upregulation of glycolysis and the predictive value of Glyc.Sig in FH-deficient RCC and pan-cancer data, we hypothesized that the hub gene of CRISPR cohorts, LDHA, might play a key role in this process. LDHA encodes the final step of glycolysis, where pyruvate is converted into lactate, generating ATP. Analysis of the FH-deficient RCC scRNA-seq dataset revealed that LDHA expression was notably higher in FH-deficient RCC epithelial cells (**Figure 7D**) compared to other cell types. Additionally, when comparing LDHA expression across metastasis, primary tumors, and adjacent normal tissues using the GSE157256 dataset (15), we found significantly lower LDHA levels in adjacent normal tissues (**Figure 7E**). To further confirm these results, immunoblotting analysis and IHC staining from patients at Renji Hospital showed significantly higher LDHA expression in tumor tissues compared to normal adjacent tissues (**Figures 7F, G**).

## Discussion

Conventional biomarker discovery has primarily relied on whole-exome sequencing (WES) or bulk-tissue RNA-seq analyses (76, 96). These methodologies are constrained by their inability to resolve cellular heterogeneity, generating population-averaged genetic profiles that obscure critical cell subtype-specific variations. The limited predictive accuracy of existing immune checkpoint inhibitor biomarkers derived from bulk analyses highlights inherent technical constraints. Breakthroughs in single-cell RNA sequencing have revolutionized biomarker discovery by enabling high-resolution transcriptomic profiling at the individual cell level (97), thereby uncovering previously undetectable molecular patterns with enhanced prognostic capabilities (98).

FH-deficient RCC, an uncommon yet aggressive malignancy, lacks established therapeutic standards, creating a critical gap in treatment options for this fatal condition. These tumors exhibit heightened immunogenicity, marked by rich lymphocyte infiltration and checkpoint protein overexpression (5), suggesting a responsive microenvironment. Recent studies report improved responses and survival rates when combining ICIs with tyrosine kinase inhibitors (TKIs) in advanced FH-deficient RCC across treatment phases, outperforming TKI monotherapy. However, objective response rates remain modest (16.7%–43.2%), indicating a need for further optimization (4, 8, 16). Loss of fumarate hydratase drives metabolic reprogramming toward aerobic glycolysis in FH-deficient RCC (99), offering insights for diagnostic strategies and therapeutic avenues.

While the interplay between tumor glycolysis and immune regulation has been extensively studied, clinical validation of the impact of glycolysis on ICI effectiveness remains limited. Through GSVA-based evaluation of single-cell glycolytic activity in malignant cells, we identified a consistent negative correlation between metabolic activity and therapeutic outcomes across four distinct ICI-treated cohorts (FH-deficient RCC, ccRCC, SKCM, and BCC). This metabolic–immune axis appears particularly significant given the established role of glycolysis as a cancer-enabling mechanism that promotes proliferation and treatment resistance in multiple tumor types (100).

Building upon these findings, we proposed that impaired immunotherapy effectiveness might universally correlate with elevated glycolytic activity in diverse malignancies. To systematically investigate this relationship, we conducted a pan-cancer analysis to identify genes that are overexpressed in malignant cells and positively associated with glycolytic flux, identifying 103 genes (Glyc.Sig). This conserved 103-gene panel demonstrated superior predictive accuracy for ICI responsiveness compared to existing biomarkers (T.cell.inflamed.Sig, INFG.Sig, PDL1.Sig, NLRP3.Sig, LRRC15.CAF.Sig, Cytotoxic.Sig) across 10 independent ICI cohorts spanning five cancer types. Our work establishes the first multi-omics validated link between tumor glycolysis and immunotherapy resistance while providing a clinically actionable predictive tool applicable across multiple cancer types, potentially for FH-RCC.

Our analysis revealed significant enrichment of Glyc.Sig genes in critical biological processes, including glycolysis, cellular response to hypoxia, HIF-1 signaling pathway, KEAP1−NFE2L2 pathway, G1/S DNA damage checkpoints, and glutathione metabolism. Hypoxic conditions stabilize HIF-1α, which transcriptionally upregulates glycolytic enzymes (e.g., LDHA, PGK1) and glucose transporters (e.g., GLUT-1). This process is synergistically amplified by interactions with c-Myc, NF-κB, and other oncogenic factors, establishing a feedforward loop to maintain elevated glycolytic flux for tumor bioenergetics (101). NFE2L2 (NRF2) dynamically balances antioxidant defense and glycolytic metabolism under stress. During oxidative stress, NFE2L2 evades KEAP1-dependent ubiquitination, accumulates in the nucleus, and activates antioxidant response element (ARE)-regulated genes. Concurrently, NFE2L2 directly enhances hexokinase 1 (HK1) expression and indirectly primes glycolysis, prioritizing ATP generation over oxidative phosphorylation (102, 103). Enhanced glycolytic flux elevates lactate synthesis, which catalyzes the lactylation of XRCC1 to promote its nuclear import, thereby augmenting DNA damage repair and fostering resistance to DNA-damaging therapies like chemoradiotherapy (104). In parallel, lactate-mediated lactylation of NBS1 stabilizes the MRE11–RAD50–NBS1 (MRN) complex, potentiating homologous recombination (HR)-dependent repair of DNA double-strand breaks (105).

Pan-cancer transcriptomic profiling from TCGA datasets demonstrated that tumors exhibiting elevated Glyc.Sig activity showed significant suppression of immune-related gene networks and decreased lymphocyte infiltration. Notably, we identified a notable inverse correlation between B-cell abundance and Glyc.Sig intensity. Given the established role of B-cell-mediated TLSs in enhancing immunotherapy efficacy through antigen presentation and T-cell priming (106), we further explored the relationship between TLS and Glyc.Sig. We revealed a significant inverse relationship between TLS scores and Glyc.Sig, suggesting that glycolytic activity may impair TLS formation. Further analysis revealed the upregulation of several immune-related biological functions, including metabolism, MYC signaling, and DNA repair. The shift toward a hypermetabolic state was implicated in evading immune surveillance (90). Elevated MYC signaling suppresses immune responses by increasing the expression of CD47 and PD-L1 (91). Enhanced DNA repair enables malignant cells to survive in hostile environments (107). These findings align with Glyc.Sig’s high-scoring tumors showing marked immunosuppression, validating its prognostic significance. Moreover, we noted a concordance between Glyc.Sig and both tumor mutational burden and intratumoral heterogeneity. Notably, high TMB is associated with increased glycolysis. Despite TMB’s predictive role for ICIs, many high-TMB patients show treatment resistance (108). Intriguingly, our stratified analysis confirmed Glyc.Sig’s inverse relation to antitumor immune function across TMB strata, implicating cancer’s glucose metabolism as a pivotal factor in high-TMB immune escape. This underscores Glyc.Sig’s pivotal role as a predictive biomarker in immunotherapy responsiveness.

Glyc.Sig emerges as an innovative biomarker, excelling in forecasting ICI responsiveness and identifying patients with survival benefits. In comparative assessments against six leading pan-cancer biomarker sets (81–85), it demonstrated superior predictive strength and adaptability across diverse cohorts. Its consistent performance highlights robustness and generalizability, outperforming existing tools. Beyond refining patient stratification, our research aims to uncover synergistic combination therapeutic strategies capable of counteracting immune evasion mechanisms. The strong association between Glyc.Sig and ICI therapeutic efficacy prompted our systematic investigation. Through computational analysis of genome-wide CRISPR screening datasets, we identified targetable vulnerabilities within Glyc.Sig-enriched tumor ecosystems, prioritizing candidates with dual potential to enhance ICI responsiveness and reverse resistance pathways. As a prominent Glyc.Sig gene, LDHA critically mediates ICI resistance by reshaping TME. Mechanistically, LDHA converts pyruvate to lactate, which subsequently undergoes lactylation to establish an inhibitory epigenetic network that broadly suppresses immune surveillance. This metabolic shift enables tumor cells to dominate nutrient utilization while generating a lactate-rich niche characterized by extracellular acidosis and systemic immunosuppression. The lactate-dominated milieu differentially modulates immune cells by impairing the cytotoxic function of CD8^+^ T cells (109), natural killer (NK/NKT) cells (11), and dendritic cells (110) through metabolic interference and inhibition of signaling pathways. Additionally, it sustains immunosuppressive phenotypes in regulatory T (Treg) cells (12) and promotes lactate-induced M2 macrophage polarization through HIF-1α stabilization, along with the induction of vascular endothelial growth factor (VEGF) and arginase-1 (ARG1) (111). Integrative analysis of pan-cancer CRISPR screening data revealed that genetic ablation of top-tier glycolysis-associated signature components—including PSMF1, GCLC, MAF1, and KLHL24—demonstrated enhanced antitumor immunity across melanoma, breast carcinoma, renal cell carcinoma, and colorectal adenocarcinoma models. These metabolic regulators emerge as promising therapeutic vulnerabilities for developing immunotherapy combination strategies through modulating immune–metabolic crosstalk in the tumor microenvironment. Additionally, they offer potential targets for FH-RCC treatment and could improve the efficacy of ICIs.

Our investigation has three principal constraints. First, the analyzed bulk ICI cohorts excluded FH-RCC cases due to the scarcity of patients with this tumor subtype, necessitating future validation of identified therapeutic targets in dedicated FH-RCC immunotherapy cohorts. Second, critical clinical parameters—including demographic variables, tumor staging, mutational burden, and intratumoral heterogeneity—were missing from certain transcriptomic datasets, limiting comprehensive survival modeling through multivariable regression. Third, while the 10 RNA-sequencing immunotherapy cohorts spanned five malignancies (gastric cancer, melanoma, renal cell carcinoma, urothelial cancer, and glioblastoma), the pan-cancer predictive capacity of Glyc.Sig requires verification in prospective trials across additional tumor types, despite compensatory evidence from multi-cancer analyses demonstrating Glyc.Sig’s inverse correlation with immune activation. Our future research will prioritize prospective clinical validation of Glyc.Sig in FH-deficient RCC cohorts receiving ICIs, coupled with functional studies targeting LDHA and other Glyc.Sig components to validate combinatorial synergies with immunotherapy. Mechanistic exploration of lactate-mediated immunosuppression—particularly lactylation-dependent epigenetic reprogramming and metabolic crosstalk impairing TLS formation—warrants deeper investigation. Integrating spatial transcriptomics will further resolve glycolytic–immune cell interactions within tumor niches.

## Conclusions

Our research marked the first robust clinical evidence linking cancer’s glucose metabolism to resistance against immune checkpoint inhibitors. By analyzing single-cell transcriptomics across various cancers, we devised Glyc.Sig, a gene expression signature that outperforms established signatures in forecasting ICI treatment responses in different patient groups. This signature not only enhances our ability to identify patients who may benefit from immunotherapy but also uncovers new therapeutic avenues, particularly highlighting LDHA as a combinatory therapeutic target for FH-deficient RCC treatment. Our findings open avenues for precision immunotherapy and propose strategies to overcome ICI resistance by targeting cancer’s sugar metabolism to enhance immune response against tumors.

## Data Availability

The original contributions presented in the study are included in the article/**Supplementary Material**. Further inquiries can be directed to the corresponding author.
