# Supplementary material for: Integrated multi-omics analysis reveals a glycolytic signature that predicts pan-cancer immune checkpoint inhibitor response and LDHA as a combinatorial target in fumarate hydratase-deficient renal cell carcinoma
Source: Front Immunol. 2025 Oct 3;16:1666121. doi: 10.3389/fimmu.2025.1666121 (PMC12531171; doi:10.3389/fimmu.2025.1666121)
Supplement: Supplementary file 1 [file DataSheet1.zip › Supplementary Figures.docx]

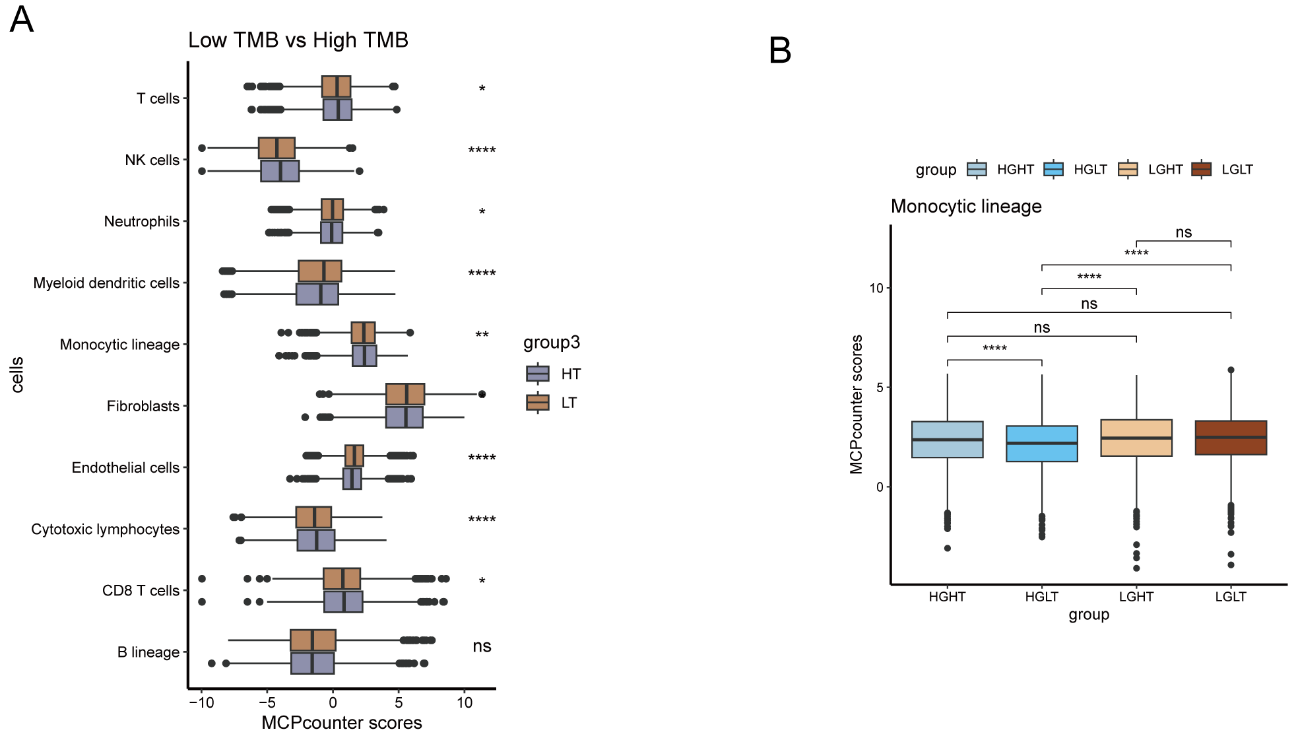


Fig. S1 Analysis of immune cell infiltration, TMB and Glyc.sig

(A) Boxplots demonstrating the associations between immune cells infiltration and TMB.

(B) Boxplots demonstrating the associations between monocytic lineage cell infiltration and Glyc.Sig.


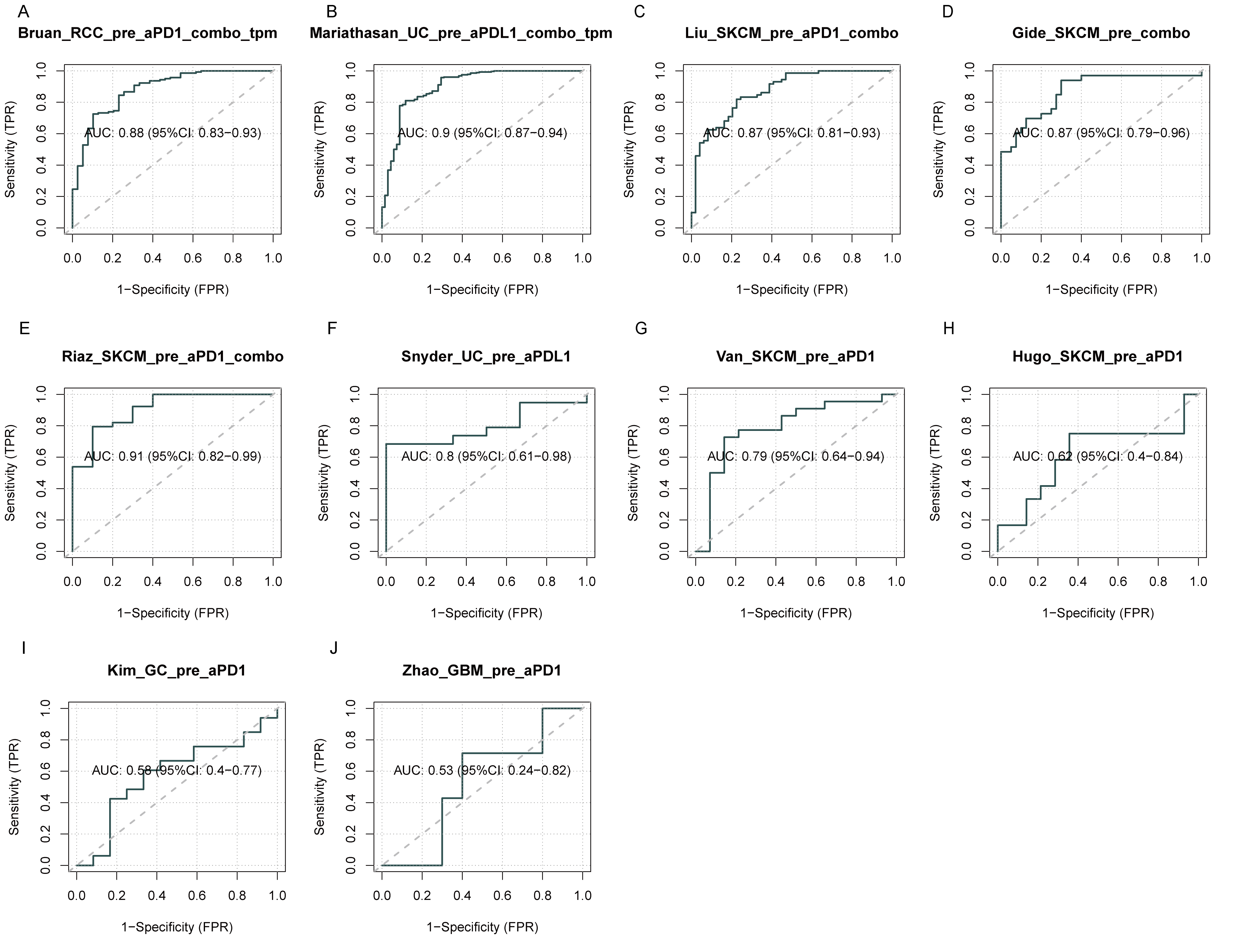


Fig. S2 ROC plot showing the performance of Glyc.Sig in individual cohort.


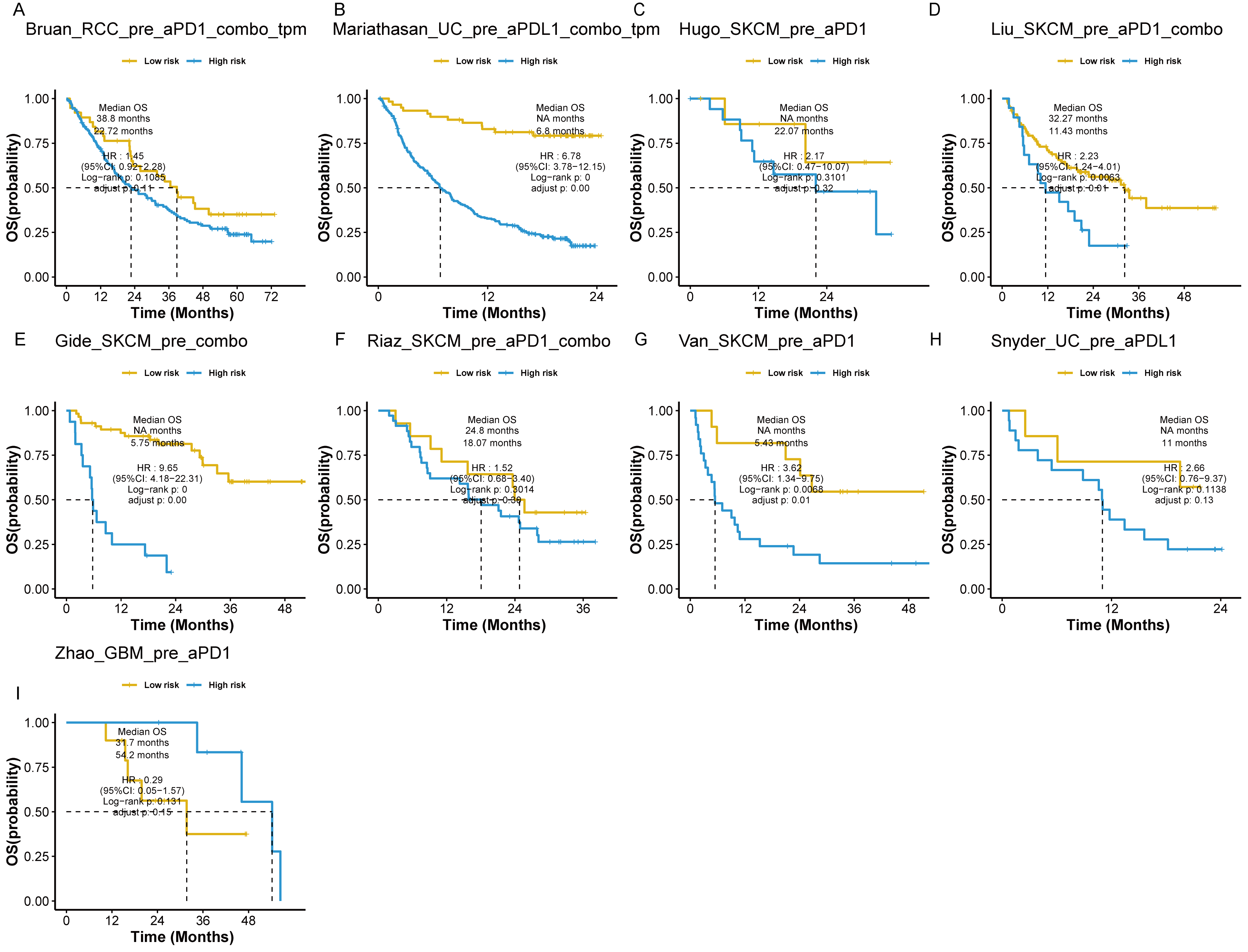


Fig. S3 Kaplan-Meier survival analysis of individual cohorts.


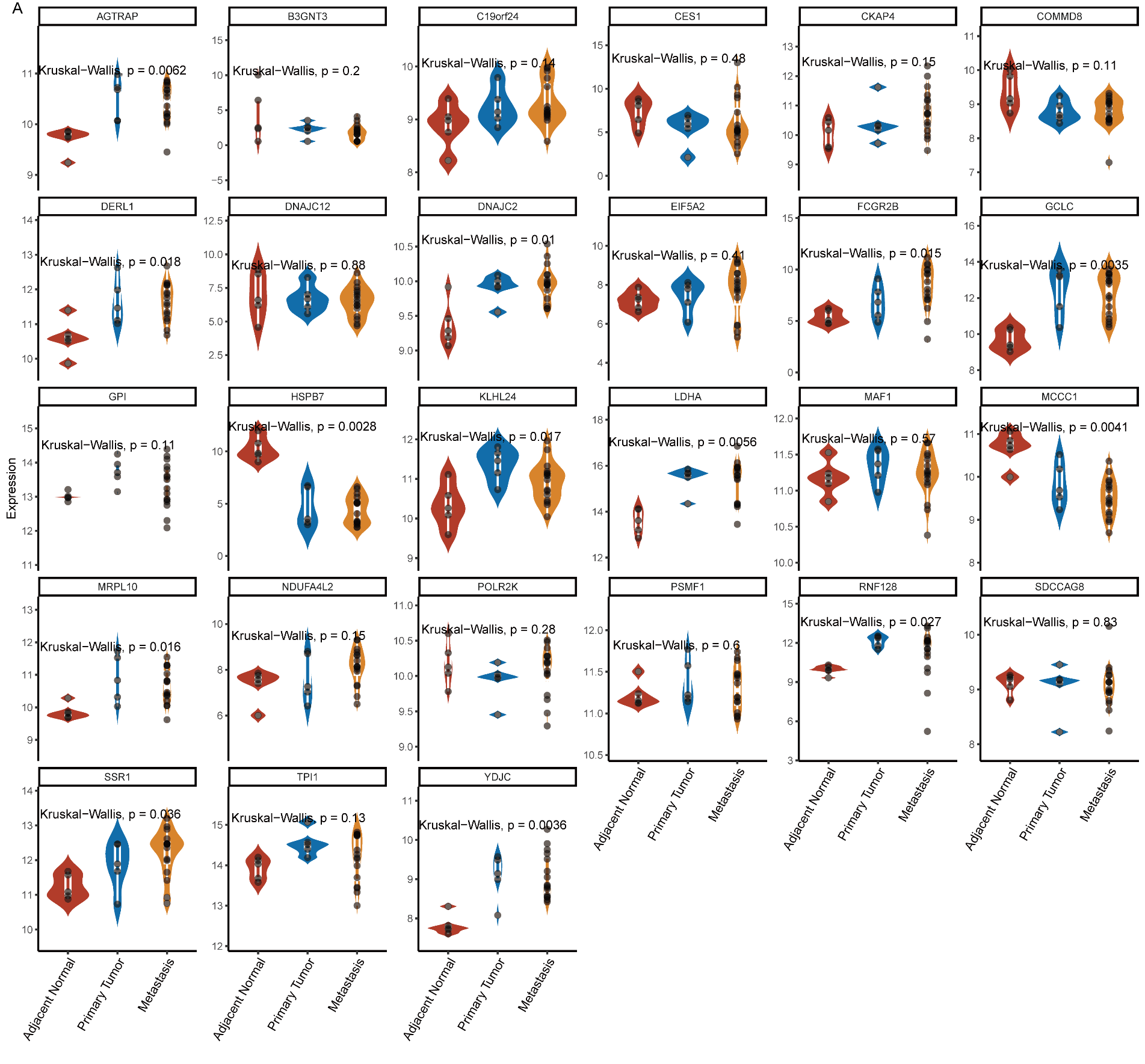


Fig. S4 Comparison among expression levels of suspicious top-ranked genes in metastasis lesion, primary lesion and adjacent normal tissue in HLRCC.
