# Supplementary material for: Integrated multi-omics analysis reveals a glycolytic signature that predicts pan-cancer immune checkpoint inhibitor response and LDHA as a combinatorial target in fumarate hydratase-deficient renal cell carcinoma
Source: Front Immunol. 2025 Oct 3;16:1666121. doi: 10.3389/fimmu.2025.1666121 (PMC12531171; doi:10.3389/fimmu.2025.1666121)
Supplement: Supplementary file 1 [file DataSheet1.zip › Supplementory Tables.docx]

**Table S1 Characteristics of patients in ICIs scRNA cohorts.**

|  | SKCM[1] | BCC[2] | ccRCC[3] | FH-deficient RCC [4] |
| --- | --- | --- | --- | --- |
| Response | 0 | 6 | 2 | 1 (PR) |
| Non-response | 11 | 4 | 1 | 3 (PD) |
| Treatment naïve | 13 | 0 | 0 | 0 |
| Overall | 24^a^ | 10^b^ | 3 | 4^c^ |
| data source | GSE115978 | GSE123813 | SRP308561 | Clin Cancer Res. |

a Seven patients have been removed for lack of malignant cells data.

b One patient has been removed for aberrant CNV profiles and mutation compared with other patients.

c Three patients have been removed for lack of information (two untreated patients and one patient without evaluation).

**Table S2. List of scRNA datasets used for the development of Glyc.Sig.**

| No. | Dataset | Cancer | Patients | Cells | PMID |
| --- | --- | --- | --- | --- | --- |
| 1 | GSE123813 [2] | BCC | 11 | 52884 | 31359002 |
| 2 | GSE143423 [5] | BRCA | 2 | 4375 | bioRxiv |
| 3 | SRP114962 [6] | BRCA | 8 | 2472 | 29681456 |
| 4 | GSE125449 [7] | CHOL | 10 | 5761 | 31588021 |
| 5 | GSE146771 [8] | CRC | 10 | 10468 | 32302573 |
| 6 | GSE102130 [9] | GBM | 6 | 3321 | 29674595 |
| 7 | GSE103224 [10] | GBM | 8 | 17185 | 30041684 |
| 8 | GSE131928a [11] | GBM | 9 | 13553 | 31327527 |
| 9 | GSE131928b [11] | GBM | 28 | 7930 | 31327527 |
| 10 | GSE138794 [12] | GBM | 9 | 18458 | 31554641 |
| 11 | GSE139448 [13] | GBM | 3 | 12152 | 32004492 |
| 12 | GSE141982 [14] | GBM | 2 | 5263 | 32105316 |
| 13 | GSE148842 [15] | GBM | 7 | 111397 | bioRxiv |
| 14 | GSE70630 [16] | GBM | 6 | 4347 | 27806376 |
| 15 | GSE84465 [17] | GBM | 4 | 3533 | 29091775 |
| 16 | GSE89567 [18] | GBM | 10 | 6341 | 28360267 |
| 17 | GSE103322 [19] | HNSC | 18 | 5902 | 29198524 |
| 18 | GSE125449 [7] | LIHC | 9 | 3834 | 31588021 |
| 19 | GSE119926 [20] | MB | 25 | 7745 | 31341285 |
| 20 | GSE117988 [21] | MCC | 1 | 10134 | 30250229 |
| 21 | GSE118056 [21] | MCC | 1 | 11024 | 30250229 |
| 22 | GSE117156 [22] | MM | 14 | 24918 | 30523328 |
| 23 | GSE140312 [23] | NET | 1 | 3158 | 32054662 |
| 24 | EMTAB6149 [24] | NSCLC | 8 | 40218 | 29988129 |
| 25 | GSE117570 [25] | NSCLC | 4 | 11453 | 31033233 |
| 26 | GSE127465 [26] | NSCLC | 7 | 31179 | 30979687 |
| 27 | GSE143423 [5] | NSCLC | 3 | 12193 | bioRxiv |
| 28 | GSE118828 [27] | OV | 9 | 1909 | 30383866 |
| 29 | CRA001160 [28] | PAAD | 35 | 57443 | 31273297 |
| 30 | GSE111672 [29] | PAAD | 3 | 6122 | 31932730 |
| 31 | GSE115978 [1] | SKCM | 31 | 7186 | 30388455 |
| 32 | GSE72056 [30] | SKCM | 19 | 4645 | 27124452 |
| 33 | GSE134520 [31] | STAD | 13 | 41554 | 31067475 |
| 34 | GSE139829 [32] | UVM | 11 | 103703 | 31980621 |
| 35 | GSE156632 [33] | ccRCC | 7 | 37243 | [35605062](https://www.ncbi.nlm.nih.gov/pubmed/35605062) |
| 36 | GSE159115 [34] | ccRCC | 7 | 26392 | [34099557](https://www.ncbi.nlm.nih.gov/pubmed/34099557) |
| 37 | GSE178481 [35] | ccRCC | 10 | 175485 | [36180422](https://www.ncbi.nlm.nih.gov/pubmed/36180422) |
| 38 | GSE207493 [36] | ccRCC | 19 | 102723 | [36607615](https://www.ncbi.nlm.nih.gov/pubmed/36607615) |
| 39 | GSE210042 [37] | ccRCC | 7 | 15000 | [37335139](https://www.ncbi.nlm.nih.gov/pubmed/37335139) |
| 40 | SRP308561 [3] | ccRCC | 6 | 167283 | 33861994 |
| 41 | Clin Cancer Res. [4] | FH-deficient RCC | 4 | 32479 | 36074152 |

**Table S3. List of immunotherapy cohorts used in this study.**

|  |  |  |  | Response | |  |  |  |  |  |
| --- | --- | --- | --- | --- | --- | --- | --- | --- | --- | --- |
| Cohorts | Cancer | Treatment | patients | R  (CR/PR) | NR  (PD/SD) |  |  |  |  |  |
| Training and Validation | | | | | |  |  |  |  |  |
| Riaz 2017 SKCM [38] | SKCM | aPD-1 | 49^b^ | 10 | 41 |  |  |  |  |  |
| Mariathasan 2018 UC [39] | UC | aPD-L1 | 348 | 68 | 280 |  |  |  |  |  |
| Braun 2020 RCC [40] | RCC | aPD-1 | 181 | 39 | 142 |  |  |  |  |  |
| Liu 2019 SKCM [41] | SKCM | aPD-1 | 121 | 47 | 74 |  |  |  |  |  |
| Gide 2019 SKCM^c^ [42] | SKCM | aPD-1 /Comb^d^ | 73 | 33 | 40 |  |  |  |  |  |
| Independent Testing | | | | | |  |  |  |  |  |
| Zhao 2019 GBM [43] | GBM | aPD-1 | 17^e^ | 10 | 7 |  |  |  |  |  |
| Van 2015 SKCM [44] | SKCM | aCTLA-4 | 36 | 14 | 22 |  |  |  |  |  |
| Kim 2018 GC^f^ [45] | GC | aPD-1 | 45 | 12 | 33 |  |  |  |  |  |
| Synder 2017 UC [46] | UC | aPD-L1 | 25 | 6 | 19 |  |  |  |  |  |
| Hugo 2016 SKCM [47] | SKCM | aPD-1 | 26^e^ | 12 | 14 |  |  |  |  |  |

a During multi-variate cox regression analysis of overall survival, patients with missing covariates data have been deleted.

b Two patients from Riaz 2017 were removed for not knowning their response.

c Gide 2019 cohort had pre-ICI biopsies for melanoma patients treated with (1) anti-PD-1 (41 patients: 22 non-responders & 19 responders) or (2) the combination of anti-PD-1 and anti-CTLA-4 drugs (32 patients: 11 non-responders & 21 responders).

d Comb: combined therapy of anti-PD-1 and anti-CTLA4.

e Zhao 2019 comprises 34 pre-treated tumor samples from 17 patients. Hugo 2016 comprises 27 pre-treated tumor samples from 26 patients.For these two cohorts, a single sample were randomly selected to represent the corresponding patient.

f Kim 2018 does not include survival data.

Abbreviation: R: responder; NR: non-responder; RCC: renal cell carcinoma; UC: Urothelial carcinoma; SKCM: skin cutaneous melanoma; GBM: glioblastoma; aPD-1: anti-PD-1 antibody; aPD-L1: anti-PD-L1 antibody; TMB: tumor mutational burden; ITH: intratumor heterogeneity

**Table S4. List of CRISPR datasets.**

| CRISPR dataset | condition | cancer | model cell |
| --- | --- | --- | --- |
| Kearney 2018 [48] | NK^1^ | Colon | MC38 |
|  | OT1+IgG^2^ | Colon | MC38 |
|  | OT1+PD1^3^ | Colon | MC38 |
| Pan 2018 [49] | OT1^4^ | Melanoma | B16 |
|  | Pmel-1_T^5^ | Melanoma | B16 |
| Manguso 2017 [50] | GVAX^6^ | Melanoma | B16 |
|  | GVAX+PD1^7^ | Melanoma | B16 |
| Vredevoogd 2019 [51] | MART1_T^8^ | Melanoma | D10 IFNGR1- |
| Patel 2017 [52] | NY-ESO-1_MART-1 _T^9^ | Melanoma | Mel624 |
| Freeman 2019 [53] | NK^1^ | Melanoma | B16 |
|  | OT1^4^ | Melanoma | B16 |
| Lawson 2020 [54] | Mid_CTL^10^ | Breast | EMT6HA |
|  | Mid_CTL^10^ | Breast | X4T1HA |
|  | Mid_CTL^10^ | Colon | CT26HA |
|  | Mid_CTL^10^ | Colon | MC38OVA |
|  | Mid_CTL^10^ | Melanoma | B16OVA |
|  | Mid_CTL^10^ | Renal | RencaHA |

1 NK: natural killer cells;

2 OT1+IgG: OT1 + immunoglobulin;

3 OT1+PD1: OT1 + anti–PD-1 antibody;

4 OT1: ovalbumin-specific transgenic CD8 T cells;

5 Pmel1_T: Pmel-1 T cells (specific for gp100 melanoma antigen);

6 GVAX: a granulocyte-macrophage colony-stimulating factor (GM-CSF)-secreting, irradiated tumor cell vaccine;

7 GVAX+PD1: GVAX + anti–PD-1 antibody;

8 MART-1_T: CD8 T cells, which had been retrovirally transduced with a MART-1-specific T cell receptor (MART-1 T

cells).

9 NY-ESO-1+_MART-1+ _T: CD8 T cells, which had been retrovirally transduced with a MART-1-specific and NY-ESO-1-

specific T cell receptor (MART-1 T cells).

10 Mid_CTL: cytotoxic cells at mid time point (defined as the population of cells following 1 or 2 treatment rounds);

**Table S5. List of predictive gene expression signatures for immunotherapy.**

| Gene signature | algorithm | brief introduction |
| --- | --- | --- |
| Glyc.Sig | Naïve Bayes | Novel stemness signature identified by large-scale single cell RNA sequencing analysis |
| IFNG.Sig [55] | Average gene expression | An IFN-gamma-related profile that predicts PD-1blockade efficacy |
| Cytotoxic.Sig [56] | Geometric mean of gene expression | Genes associated with cytotoxic activities |
| NLRP3.Sig [57] | ssGSEA | NLRP3 inflammasome-related genes derived from pan-cancer analysis |
| LRRC15.CAF.Sig [58] | eigenWeightedMean^a^ | Gene expression signature of LRRC15+ Cancer associated fibroblast |
| T.cell.inflamed.Sig [55] | Average gene expression | T-cell inflamed gene expression profiles |
| PD-L1.Sig [59] | Expression of PD-L1 | Gene expression profiles of PD-L1 / PDCD1. |

a provided by R package multiGSEA v1.1.99

**Table S6. Gene list of Stem.Sig.**

| AGTR1 | PPP4C | NTS | GPI | PKP1 |
| --- | --- | --- | --- | --- |
| AC007325.4 | PGAM1 | TSPAN8 | RNF128 | LDHA |
| COMMD8 | MAD2L1BP | GPX2 | AKR1C2 | IMPAD1 |
| VCX2 | IGKC | MIR205HG | POLR1C | GCLC |
| POLR2G | TPD52L1 | G6PD | CEACAM6 | KRT6B |
| ENO2 | PSMD13 | JCHAIN | SRSF6 | POLR2K |
| CCT6P3 | RP11-295G20.2 | MGC39584 | COL22A1 | PCOLCE2 |
| SDCCAG8 | NAA50 | COMMD7 | PTDSS1 | MAF1 |
| YDJC | C19orf24 | CES1 | AKR1B10 | NMT1 |
| B3GNT3 | SF3B3 | CA9 | DNAJC12 | C6ORF62 |
| UBAC2 | PSMC6 | NUTF2 | FCGR2B | NDUFA4L2 |
| AGR2 | AGTRAP | MPZL1 | TMEM99 | MRPL10 |
| COL7A1 | CKAP4 | DERL1 | PSMF1 | OSGIN1 |
| TAF10 | GALNT1 | HAX1 | TMEM66 | PFKP |
| NUP37 | LINC01419 | GOT2 |  |  |

**Table S7. Comparison of AUC of previous signatures in testing cohort.**

| Pan-cancer signatures | Glyc.Sig | INFG.Sig | 1. cell.   inflamed.Sig | Cytotoxic.Sig | | PDL1.Sig | | LRRC15.CAF.Sig | NLRP3.Sig |
| --- | --- | --- | --- | --- | --- | --- | --- | --- | --- |
| validation | 0.69 | 0.54 | 0.51 | 0.40 | 0.50 | | 0.41 | | 0.55 |
| testing | 0.66 | 0.66 | 0.65 | 0.48 | 0.62 | | 0.60 | | 0.60 |
| Bruan RCC | 0.88 | 0.48 | 0.52 | 0.51 | 0.51 | | 0.43 | | 0.56 |
| Mariathasan UC | 0.90 | 0.62 | 0.53 | 0.57 | 0.56 | | 0.52 | | 0.48 |
| Hugo SKCM | 0.62 | 0.53 | 0.57 | 0.43 | 0.52 | | 0.60 | | 0.46 |
| Liu SKCM | 0.87 | 0.52 | 0.52 | 0.61 | 0.48 | | 0.51 | | 0.53 |
| Gide SKCM | 0.87 | 0.83 | 0.77 | 0.19 | 0.76 | | 0.48 | | 0.73 |
| Riaz SKCM | 0.91 | 0.64 | 0.63 | 0.59 | 0.66 | | 0.53 | | 0.59 |
| Van SKCM | 0.79 | 0.67 | 0.73 | 0.29 | 0.69 | | 0.52 | | 0.67 |
| Kim GC | 0.58 | 0.85 | 0.78 | 0.77 | 0.79 | | 0.55 | | 0.62 |
| Zhao GBM | 0.53 | 0.53 | 0.51 | 0.57 | 0.46 | | 0.63 | | 0.74 |
| Snyder UC | 0.80 | 0.54 | 0.60 | 0.49 | 0.51 | | 0.81 | | 0.46 |
| mean | 0.76 | 0.62 | 0.61 | 0.49 | 0.59 | | 0.55 | | 0.58 |

References

1. Jerby-Arnon, L., et al., *A Cancer Cell Program Promotes T Cell Exclusion and Resistance to Checkpoint Blockade.* Cell, 2018. **175**(4): p. 984-997.e24.

2. Yost, K.E., et al., *Clonal replacement of tumor-specific T cells following PD-1 blockade.* Nat Med, 2019. **25**(8): p. 1251-1259.

3. Krishna, C., et al., *Single-cell sequencing links multiregional immune landscapes and tissue-resident T cells in ccRCC to tumor topology and therapy efficacy.* Cancer Cell, 2021. **39**(5): p. 662-677.e6.

4. Dong, P., et al., *Genomic Characteristics and Single-Cell Profiles After Immunotherapy in Fumarate Hydratase-Deficient Renal Cell Carcinoma.* Clin Cancer Res, 2022. **28**(21): p. 4807-4819.

5. Wang, L., et al., *Single-Cell Map of Diverse Immune Phenotypes in the Metastatic Brain Tumor Microenvironment of Non Small Cell Lung Cancer.* bioRxiv, 2019: p. 2019.12.30.890517.

6. Kim, C., et al., *Chemoresistance Evolution in Triple-Negative Breast Cancer Delineated by Single-Cell Sequencing.* Cell, 2018. **173**(4): p. 879-893.e13.

7. Ma, L., et al., *Tumor Cell Biodiversity Drives Microenvironmental Reprogramming in Liver Cancer.* Cancer Cell, 2019. **36**(4): p. 418-430.e6.

8. Zhang, L., et al., *Single-Cell Analyses Inform Mechanisms of Myeloid-Targeted Therapies in Colon Cancer.* Cell, 2020. **181**(2): p. 442-459.e29.

9. Filbin, M.G., et al., *Developmental and oncogenic programs in H3K27M gliomas dissected by single-cell RNA-seq.* Science, 2018. **360**(6386): p. 331-335.

10. Yuan, J., et al., *Single-cell transcriptome analysis of lineage diversity in high-grade glioma.* Genome Med, 2018. **10**(1): p. 57.

11. Neftel, C., et al., *An Integrative Model of Cellular States, Plasticity, and Genetics for Glioblastoma.* Cell, 2019. **178**(4): p. 835-849.e21.

12. Wang, L., et al., *The Phenotypes of Proliferating Glioblastoma Cells Reside on a Single Axis of Variation.* Cancer Discov, 2019. **9**(12): p. 1708-1719.

13. Wang, R., et al., *Adult Human Glioblastomas Harbor Radial Glia-like Cells.* Stem Cell Reports, 2020. **14**(2): p. 338-350.

14. Wang, L., et al., *Ensemble learning for classifying single-cell data and projection across reference atlases.* Bioinformatics, 2020. **36**(11): p. 3585-3587.

15. Zhao, W., et al., *Deconvolution of Cell Type-Specific Drug Responses in Human Tumor Tissue with Single-Cell RNA-seq.* bioRxiv, 2020: p. 2020.04.22.056341.

16. Tirosh, I., et al., *Single-cell RNA-seq supports a developmental hierarchy in human oligodendroglioma.* Nature, 2016. **539**(7628): p. 309-313.

17. Darmanis, S., et al., *Single-Cell RNA-Seq Analysis of Infiltrating Neoplastic Cells at the Migrating Front of Human Glioblastoma.* Cell Rep, 2017. **21**(5): p. 1399-1410.

18. Venteicher, A.S., et al., *Decoupling genetics, lineages, and microenvironment in IDH-mutant gliomas by single-cell RNA-seq.* Science, 2017. **355**(6332).

19. Puram, S.V., et al., *Single-Cell Transcriptomic Analysis of Primary and Metastatic Tumor Ecosystems in Head and Neck Cancer.* Cell, 2017. **171**(7): p. 1611-1624.e24.

20. Hovestadt, V., et al., *Resolving medulloblastoma cellular architecture by single-cell genomics.* Nature, 2019. **572**(7767): p. 74-79.

21. Paulson, K.G., et al., *Acquired cancer resistance to combination immunotherapy from transcriptional loss of class I HLA.* Nat Commun, 2018. **9**(1): p. 3868.

22. Ledergor, G., et al., *Single cell dissection of plasma cell heterogeneity in symptomatic and asymptomatic myeloma.* Nat Med, 2018. **24**(12): p. 1867-1876.

23. Rao, M., et al., *Comparative single-cell RNA sequencing (scRNA-seq) reveals liver metastasis-specific targets in a patient with small intestinal neuroendocrine cancer.* Cold Spring Harb Mol Case Stud, 2020. **6**(2).

24. Lambrechts, D., et al., *Phenotype molding of stromal cells in the lung tumor microenvironment.* Nat Med, 2018. **24**(8): p. 1277-1289.

25. Song, Q., et al., *Dissecting intratumoral myeloid cell plasticity by single cell RNA-seq.* Cancer Med, 2019. **8**(6): p. 3072-3085.

26. Zilionis, R., et al., *Single-Cell Transcriptomics of Human and Mouse Lung Cancers Reveals Conserved Myeloid Populations across Individuals and Species.* Immunity, 2019. **50**(5): p. 1317-1334.e10.

27. Shih, A.J., et al., *Identification of grade and origin specific cell populations in serous epithelial ovarian cancer by single cell RNA-seq.* PLoS One, 2018. **13**(11): p. e0206785.

28. Peng, J., et al., *Single-cell RNA-seq highlights intra-tumoral heterogeneity and malignant progression in pancreatic ductal adenocarcinoma.* Cell Res, 2019. **29**(9): p. 725-738.

29. Moncada, R., et al., *Integrating microarray-based spatial transcriptomics and single-cell RNA-seq reveals tissue architecture in pancreatic ductal adenocarcinomas.* Nat Biotechnol, 2020. **38**(3): p. 333-342.

30. Tirosh, I., et al., *Dissecting the multicellular ecosystem of metastatic melanoma by single-cell RNA-seq.* Science, 2016. **352**(6282): p. 189-96.

31. Zhang, P., et al., *Dissecting the Single-Cell Transcriptome Network Underlying Gastric Premalignant Lesions and Early Gastric Cancer.* Cell Rep, 2019. **27**(6): p. 1934-1947.e5.

32. Durante, M.A., et al., *Single-cell analysis reveals new evolutionary complexity in uveal melanoma.* Nat Commun, 2020. **11**(1): p. 496.

33. Zhang, M., et al., *Single cell analysis reveals intra-tumour heterogeneity, microenvironment and potential diagnosis markers for clear cell renal cell carcinoma.* Clin Transl Med, 2022. **12**(5): p. e713.

34. Zhang, Y., et al., *Single-cell analyses of renal cell cancers reveal insights into tumor microenvironment, cell of origin, and therapy response.* Proc Natl Acad Sci U S A, 2021. **118**(24).

35. Alchahin, A.M., et al., *A transcriptional metastatic signature predicts survival in clear cell renal cell carcinoma.* Nat Commun, 2022. **13**(1): p. 5747.

36. Yu, Z., et al., *Integrative Single-Cell Analysis Reveals Transcriptional and Epigenetic Regulatory Features of Clear Cell Renal Cell Carcinoma.* Cancer Res, 2023. **83**(5): p. 700-719.

37. Davidson, G., et al., *Mesenchymal-like Tumor Cells and Myofibroblastic Cancer-Associated Fibroblasts Are Associated with Progression and Immunotherapy Response of Clear Cell Renal Cell Carcinoma.* Cancer Res, 2023. **83**(17): p. 2952-2969.

38. Riaz, N., et al., *Tumor and Microenvironment Evolution during Immunotherapy with Nivolumab.* Cell, 2017. **171**(4): p. 934-949.e16.

39. Mariathasan, S., et al., *TGFβ attenuates tumour response to PD-L1 blockade by contributing to exclusion of T cells.* Nature, 2018. **554**(7693): p. 544-548.

40. Braun, D.A., et al., *Interplay of somatic alterations and immune infiltration modulates response to PD-1 blockade in advanced clear cell renal cell carcinoma.* Nat Med, 2020. **26**(6): p. 909-918.

41. Liu, D., et al., *Integrative molecular and clinical modeling of clinical outcomes to PD1 blockade in patients with metastatic melanoma.* Nat Med, 2019. **25**(12): p. 1916-1927.

42. Gide, T.N., et al., *Distinct Immune Cell Populations Define Response to Anti-PD-1 Monotherapy and Anti-PD-1/Anti-CTLA-4 Combined Therapy.* Cancer Cell, 2019. **35**(2): p. 238-255.e6.

43. Zhao, J., et al., *Immune and genomic correlates of response to anti-PD-1 immunotherapy in glioblastoma.* Nat Med, 2019. **25**(3): p. 462-469.

44. Van Allen, E.M., et al., *Genomic correlates of response to CTLA-4 blockade in metastatic melanoma.* Science, 2015. **350**(6257): p. 207-211.

45. Kim, S.T., et al., *Comprehensive molecular characterization of clinical responses to PD-1 inhibition in metastatic gastric cancer.* Nat Med, 2018. **24**(9): p. 1449-1458.

46. Snyder, A., et al., *Contribution of systemic and somatic factors to clinical response and resistance to PD-L1 blockade in urothelial cancer: An exploratory multi-omic analysis.* PLoS Med, 2017. **14**(5): p. e1002309.

47. Hugo, W., et al., *Genomic and Transcriptomic Features of Response to Anti-PD-1 Therapy in Metastatic Melanoma.* Cell, 2016. **165**(1): p. 35-44.

48. Kearney, C.J., et al., *Tumor immune evasion arises through loss of TNF sensitivity.* Sci Immunol, 2018. **3**(23).

49. Pan, D., et al., *A major chromatin regulator determines resistance of tumor cells to T cell-mediated killing.* Science, 2018. **359**(6377): p. 770-775.

50. Manguso, R.T., et al., *In vivo CRISPR screening identifies Ptpn2 as a cancer immunotherapy target.* Nature, 2017. **547**(7664): p. 413-418.

51. Vredevoogd, D.W., et al., *Augmenting Immunotherapy Impact by Lowering Tumor TNF Cytotoxicity Threshold.* Cell, 2019. **178**(3): p. 585-599.e15.

52. Patel, S.J., et al., *Identification of essential genes for cancer immunotherapy.* Nature, 2017. **548**(7669): p. 537-542.

53. Freeman, A.J., et al., *Natural Killer Cells Suppress T Cell-Associated Tumor Immune Evasion.* Cell Rep, 2019. **28**(11): p. 2784-2794.e5.

54. Lawson, K.A., et al., *Functional genomic landscape of cancer-intrinsic evasion of killing by T cells.* Nature, 2020. **586**(7827): p. 120-126.

55. Ayers, M., et al., *IFN-γ-related mRNA profile predicts clinical response to PD-1 blockade.* J Clin Invest, 2017. **127**(8): p. 2930-2940.

56. Rooney, M.S., et al., *Molecular and genetic properties of tumors associated with local immune cytolytic activity.* Cell, 2015. **160**(1-2): p. 48-61.

57. Ju, M., et al., *Pan-cancer analysis of NLRP3 inflammasome with potential implications in prognosis and immunotherapy in human cancer.* Brief Bioinform, 2021. **22**(4).

58. Dominguez, C.X., et al., *Single-Cell RNA Sequencing Reveals Stromal Evolution into LRRC15(+) Myofibroblasts as a Determinant of Patient Response to Cancer Immunotherapy.* Cancer Discov, 2020. **10**(2): p. 232-253.

59. Topalian, S.L., et al., *Safety, activity, and immune correlates of anti-PD-1 antibody in cancer.* N Engl J Med, 2012. **366**(26): p. 2443-54.
